# Targeting ATF6α Attenuates UVB‐Induced Senescence and Improves Skin Homeostasis by Regulating IL8 Expression

**DOI:** 10.1111/acel.70024

**Published:** 2025-04-16

**Authors:** Joëlle Giroud, Pauline Delvaux, Laura Carlier, Clémentine De Schutter, Nathalie Martin, Raphaël Rouget, Ayeh Bolouki, Valérie De Glas, Inès Bouriez, Florent Bourdoux, Sophie Burteau, Julien Théry, Gauthier Decanter, Nicolas Penel, Yvan de Launoit, Benjamin Ledoux, Corinne Abbadie, Yves Poumay, Olivier Pluquet, Florence Debacq‐Chainiaux

**Affiliations:** ^1^ Laboratory of Biochemistry and Cell Biology (URBC), Namur Research Institute for Life Sciences (NARILIS) University of Namur Namur Belgium; ^2^ University of Lille, CNRS, Inserm, Pasteur Institute of Lille, UMR9020‐U1277‐CANTHER‐Cancer Heterogeneity Plasticity and Resistance to Therapies Lille France; ^3^ University of Lorraine, CNRS, CRAN‐UMR7039 Vandoeuvre‐lès‐Nancy France; ^4^ Research Unit of Molecular Physiology (URPhyM), Namur Research Institute for Life Sciences (NARILIS) University of Namur Namur Belgium; ^5^ Direction of Clinical Research and Innovation Oscar Lambret Center Lille France

**Keywords:** ATF6α, normal human dermal fibroblasts, skin, UPR, UVB‐induced senescence

## Abstract

Skin aging is influenced by both intrinsic and extrinsic factors, particularly UV radiation, and is characterized by an accumulation of senescent cells. Remarkably, exposure to UV can trigger senescence in different skin cell types, including dermal fibroblasts. However, the molecular mechanisms underlying UV‐induced senescence and the impact of the related senescence‐associated secretory phenotype (SASP) on the homeostasis of the overlying epidermis remain poorly understood. Here, we identified that both chronological aging and photoaging induce the unfolded protein response (UPR) in human dermal samples. We demonstrated that silencing ATF6α disrupts the establishment of the UVB‐induced senescent phenotype by preventing the onset of several senescent biomarkers and alters the composition of the SASP, consequently affecting its impact on the increased proliferation of keratinocytes embedded in reconstructed human epidermis. Moreover, we found that ATF6α partially mediates IL8 expression involved in the hyperproliferation of cultured keratinocytes. Together, our findings highlight the importance of the ATF6α/IL8 axis in regulating the homeostasis of neighboring cells during skin photoaging, thus suggesting ATF6α as a potentially promising target for senotherapeutic interventions.

## Graphical abstract

UVB exposure can trigger senescence in dermal fibroblasts. We show that silencing ATF6α disrupts the establishment of the UVB‐induced senescent phenotype by preventing the onset of several senescent biomarkers and altering the composition of the SASP, consequently affecting its impact on the skin microenvironment.

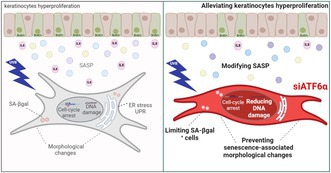

**Abbreviations**
AK: Actinic keratoses
BP: Biological process
CC: Cellular component
CM: Conditioned media
ECM: Extracellular matrix
ER: Endoplasmic reticulum
GO: Gene ontology
GSEA: gene set enrichment analysis
MF: Molecular function
MMPs: Matrix metalloproteinases
NB: Narrowband
NHDFs: Normal human dermal fibroblasts
PCA: Principal component analysis
RHE: Reconstructed human epidermis
RNA‐seq: RNA sequencing
SASP: Senescence‐associated secretory phenotype
SA‐βgal: Senescence‐associated β‐galactosidase activity
SCC: Squamous cell carcinoma
SIPS: Stress‐induced premature senescence
UPR: Unfolded protein response
UV: Ultraviolet

## 1 Introduction

Aging is a complex process characterized by numerous changes within an organism, resulting in the gradual and cumulative decline of biological functions. Aging is also associated with an increased risk of developing age‐related diseases (Childs et al. 2017). Several hallmarks, which are often interconnected, have been proposed to define aging. These include chronic inflammation, genomic instability, loss of proteostasis, altered intercellular communication, epigenetic alterations, and cellular senescence (López‐Otín et al. 2023). Over the years, the skin undergoes structural and morphological changes, leading to the deterioration of its functions (Russell‐Goldman and Murphy 2020). Skin aging is associated with both intrinsic and extrinsic factors, including sun exposure, a phenomenon known as photoaging, primarily attributable to ultraviolet (UV) rays (Kammeyer and Luiten 2015; Krutmann et al. 2017). The main effects of UV exposure on skin aging include epidermal thickening, wrinkles, solar elastosis, a decreased amount of extracellular matrix (ECM) proteins, and an increased collagen fragmentation due to elevated activity of matrix metalloproteinases (MMPs) (Wlaschek et al. 2001). Interestingly, acute UV exposure may primarily induce short‐term damage and changes in the skin, such as erythema. In contrast, chronic UV exposure may trigger long‐term damage, such as photoaging and photocarcinogenesis. Furthermore, UV radiation is a major risk factor for the development of precancerous skin lesions (actinic keratosis and naevi) and skin cancers (Narayanan et al. 2010), including melanoma and nonmelanoma skin cancers, which represent the human cancers with the highest incidence (D'Orazio et al. 2013).

Cellular senescence is a complex cellular state that can be considered a stress response phenotype and is viewed as a critical factor in both the normal aging process and aging‐associated pathologies. Senescence was initially associated with replicative lifespan exhaustion and later linked to telomere shortening, a phenomenon referred to as replicative senescence (Hayflick and Moorhead 1961; Zhu et al. 2019; Di Micco et al. 2021). Additionally, as an adaptative stress response, senescence can also be triggered following telomere‐independent mechanisms, including oncogene expression, mitochondrial dysfunction, or exposure to various stresses like ultraviolet radiation (Gorgoulis et al. 2019; Wiley et al. 2016; Di Micco et al. 2021). Despite their likely different origins, senescent cells share common features, such as a stable and strong cell cycle arrest that mainly depends on the activation of p53/p21^WAF1^ and/or p16^INK4^/pRB pathways in an interconnected or independent manner. Additionally, they exhibit senescence‐associated β‐galactosidase activity (SA‐βgal), enlarged morphology, persistent DNA damage, and a specific inflammatory secretome referred to as the senescence‐associated secretory phenotype or SASP (Hernandez‐Segura et al. 2018). The composition of SASP can vary depending on the context, although some secreted factors, such as interleukins and cytokines (e.g., IL6, IL8, CXCL1, CXCL2, CXCL3) are frequently present (Giroud et al. 2023; Gorgoulis et al. 2019).

Several studies have demonstrated that preventing the accumulation of senescent cells in aging tissues, by eliminating them with senolytics, improves tissue function and delays various age‐related diseases (Baar et al. 2017; Jeon et al. 2017; Ogrodnik et al. 2017). These findings suggest senescent cells as potential therapeutic targets, leading to another strategy to slow down the aging phenotype: the use of compounds known as senomorphics. Indeed, senomorphics suppress the detrimental effects of SASP without inducing the death of senescent cells (Zhang et al. 2023). To date, senomorphics are mainly polyphenols, and their biological effects are multiple. However, their impacts on the SASP remain largely unexplored and are mostly limited to some in vitro studies (Giroud et al. 2023).

Senescence can be induced by acute and chronic UV exposures, which differentially affect the level of UV‐induced damage, as well as the ability of cells to repair this damage. For instance, repeated UVB exposures can induce senescence in dermal fibroblasts, melanocytes, and keratinocytes (Debacq‐Chainiaux et al. 2005; Martic et al. 2020; Bauwens et al. 2023). In vivo, the build‐up of senescent cells is detected in the epidermis and dermis with age (Dimri et al. 1995; Ressler et al. 2006; Wang et al. 2017; Tuttle et al. 2020; Ogata et al. 2023). Additionally, the accumulation of senescent cells in the skin has been associated with skin aging characteristics (Waaijer et al. 2016). The detrimental effects of senescent cells can be mainly attributed to their secretome (Coppé et al. 2010), notably because of their ability to influence their microenvironment (Acosta et al. 2013; Ogata et al. 2021). For example, in the skin, senescent melanocytes have been shown to affect keratinocyte proliferation (Victorelli et al. 2019). Besides, keratinocytes exposed to conditioned media from oncogene‐induced senescent keratinocytes exhibited increased clonogenic capacity, as well as in vivo regenerative capacity (Ritschka et al. 2017).

The Endoplasmic Reticulum (ER) is the primary secretory compartment, responsible for the folding and maturation of secreted proteins. Conditions that promote the accumulation of misfolded proteins or aggregates within the ER lumen, such as nutrient deprivation, oxidative stress, or hypoxia, disrupt ER functions and lead to a state referred to as ER stress. To overcome these challenges and maintain ER homeostasis, cells set up adaptive mechanisms, including the Unfolded Protein Response (UPR). Despite profound changes in the composition of the secretome in senescent cells, there is limited understanding about how the ER adapts and copes with these changes. Interestingly, there is evidence that different cell types, when undergoing senescence, display UPR activation (Denoyelle et al. 2006; Dörr et al. 2013; Blazanin et al. 2017; Kim et al. 2019; Sabath et al. 2020). The UPR pathway is initiated by three transmembrane sensors, the activating transcription factor 6 (ATF6α), the PKR‐like ER kinase (PERK), and the Inositol Requiring Enzyme 1 (IRE1α). These sensors initiate signaling cascades that inhibit protein synthesis, upregulate chaperones and folding enzymes, and induce the degradation of misfolded proteins. However, the three branches are not always activated together at senescence, and the specific activation of one pathway varies depending on the cell type and the senescence stimuli (Matos et al. 2015; Abbadie and Pluquet 2020). We and others have demonstrated that in replicative senescence, UVC‐induced senescence, and in HRas‐induced senescence, the disruption of ATF6α alters the senescent phenotype in different skin cell types (Druelle et al. 2016; Drullion et al. 2018; Kim et al. 2019; Denoyelle et al. 2006). Indeed, ATF6α silencing in replicative senescent normal human dermal fibroblasts (NHDFs) causes the reversal of some characteristics of the senescence phenotype, such as SA‐βgal, morphology, and SASP composition (Druelle et al. 2016). Nonetheless, the significance of ATF6α in driving UVB‐induced senescence in NHDFs, as well as its role in determining SASP composition and the associated paracrine effects, remains poorly understood.

Therefore, we investigated whether the ATF6α branch of the UPR plays a role in the UVB‐induced senescent phenotype of NHDFs and examined whether the ATF6α‐dependent SASP influences the homeostasis of skin cells. Overall, our data revealed a novel role for ATF6α in the regulation of UVB‐induced senescence and skin homeostasis by influencing the behavior of neighboring cells through the paracrine control of SASP factors such as IL8.

## 2 Results

### 2.1 UPR Is Triggered During Normal Dermal Aging and Is Enhanced by Chronic Sun Exposure

**FIGURE 1:**
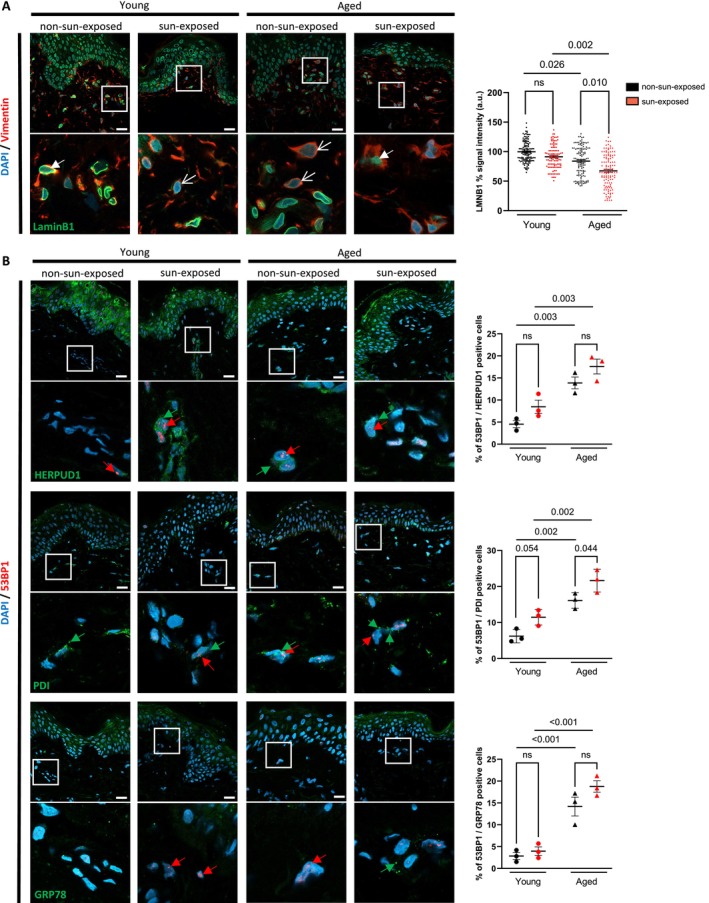
Detection of ER stress/UPR markers co‐expressed with the DNA damage regulator 53BP1 during human dermal aging and photoaging. (A) (Left panel) Histological imaging of the dermis from both young (19–39 years old) and aged donors (56–82 years old) nonsun‐exposed or sun‐exposed. Samples were stained using immunofluorescence targeting LaminB1 (green). Vimentin (red) was used to delineate the dermal regions and DAPI (blue) for nuclei. Scale bar indicates 20 μm. (Right panel) Quantifications of the percentage of LaminB1 signal intensity (*n* = 3). The loss of LaminB1 (hollow white arrowheads) and the presence of LaminB1 (full white arrowheads) are pointed. (B) (Left panel) Samples were stained using immunofluorescence targeting HERPUD1, PDI, or GRP78 (green). 53BP1 (red) was used to identify senescent cells and DAPI (blue) to stain nuclei. Scale bar indicates 20 μm. (Right panel) Quantification of the percentage of 53BP1‐positive cells co‐expressing ER stress/UPR markers (*n* = 3). 53BP1 foci (red arrowheads) and ER stress/UPR staining (green arrowheads) are pointed out. Data information: Data in (A, B) are presented as means ± SEM of three donors per group. Statistical comparisons were performed using ANOVA2 followed by Šidak's multiple comparison tests. *p*‐values shown represent statistical difference between nonsun‐exposed (NSE) and sun‐exposed (SE) dermis samples, and difference between the young and aged groups under the same sun exposure condition.

To search for a potential activation of the UPR during human skin aging, we established a collection of skin biopsies obtained from both nonsun‐exposed and sun‐exposed human dermal samples, encompassing both young and elderly donors (Table 1). To visualize the accumulation of senescent cells in the aged dermis, we performed immunostainings against LaminB1 and 53BP1, a regulator of the DNA damage response (Figure 1A and Figure S1A). Indeed, 53BP1 foci have been reported as a reliable in vivo biomarker of senescence in the skin (Nassour et al. 2016; Bauwens et al. 2023), while the loss of LaminB1 is recognized as a robust biomarker of senescent cells in photoaged skin (A. S. Wang et al. 2017). In addition, vimentin staining was used to identify dermal fibroblasts. Expectedly, LaminB1 levels were reduced in the dermis of aged human donors compared to young donors, and sun exposure further exacerbated the loss of LaminB1 in the dermis (Figure 1A). In addition, < 5% of dermal fibroblasts in young nonsun‐exposed dermis displayed 53BP1 foci, while 12% of young sun‐exposed dermis did. Moreover, sun exposure significantly increased the percentage of 53BP1 foci from 15% to 24% in fibroblasts from nonsun‐exposed compared to sun‐exposed sections in elderly donors (Figure S1A). To investigate whether UPR activation is modulated by age and sun exposure, we analyzed the percentage of positive cells for proteins involved in ER stress (PDI) and UPR (HERPUD1, XBP1s, and GRP78) in dermal sections (Figure S1B,C). Analyses of HERPUD1 and XBP1s showed a significant increase in their abundance in dermal fibroblasts from aged donors compared to young donors in both nonsun‐exposed and sun‐exposed conditions, while the proportion of PDI‐positive fibroblasts with age significantly increased only in sun‐exposed areas. Additionally, sun exposure significantly increased HERPUD1‐positive fibroblasts in skin samples from young donors (Figure S1B). To further address the question, we examined the co‐expression of cellular senescence and ER stress/UPR markers. To this end, the percentage of 53BP1‐positive cells co‐expressing HERPUD1, PDI, or GRP78 was quantified in dermal sections. A statistically significant increase in the proportion of double‐positive 53BP1 and HERPUD1 or GRP78 dermal fibroblasts was observed in the dermis of aged donors compared to young donors in both nonsun‐exposed and sun‐exposed conditions. Moreover, sun exposure strongly increased the co‐expression of 53BP1 and PDI in fibroblasts from both young and aged donors (Figure 1B).

**TABLE 1:** Characteristics of skin sample donors.

Conditions	Y_NSE	Y_SE	A_NSE	A_SE
Donors age	28.3 ± 10.1	31.7 ± 1.5	79.7 ± 2.1	66.7 ± 11
Sample localization	Right thigh, left wrist, right thigh	Right forearm, Left elbow, right forearm	Left thigh, right shoulder, right buttock	Right arm, neck, neck
Fitzpatrick type	III, III, III	IV, II, III	I, III, V	IV, II, III

*Note:* For further details, please refer to the Materials and Methods section.

Abbreviations: A, aged; NSE, nonsun‐exposed; SE, sun exposed; Y, young.

Together, these results suggest that dermal (photo)aging may involve the activation of UPR‐induced proteins.

### 2.2 Repeated UVB Exposures Induce Premature Senescence in Normal Human Dermal Fibroblasts

**FIGURE 2:**
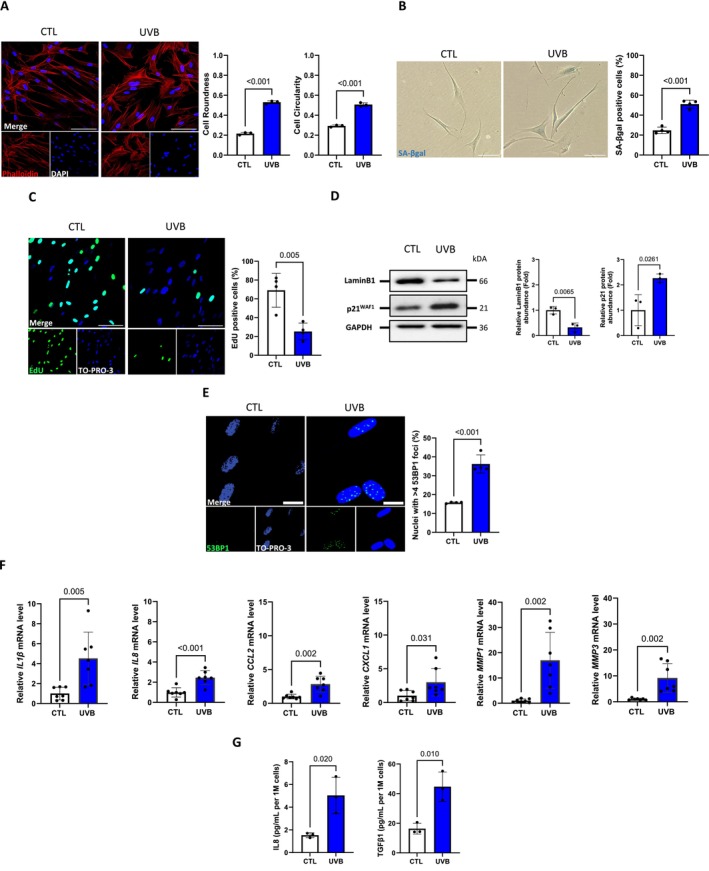
Repeated UVB exposures induce premature senescence in NHDFs. NHDFs were exposed (UVB) or not (CTL) to 500 mJ/cm^2^ UVB twice a day for 5 days. At 3 days after the last UVB exposure, the biomarkers of senescence were studied. (A) (Left panel) Representative micrographs of phalloidin (red) and TO‐PRO‐3 (blue). Scale bar indicates 100 μm. (Right panel) Quantification of cells roundness (4*area/(π*major_axis^2^)) and cell circularity (4π*area/perimeter^2^). Circularity measures the deviation from a perfect circle (Cl = 1) whereas roundness approximates a “best‐fit” to an idealized ellipse (Ro = 1). For each condition, 50 cells have been counted (*n* = 3). (B) (Left panel) Representative micrographs of SA‐βgal staining (blue). Scale bar indicates 100 μm. (Right panel) Quantification of SA‐βgal‐positive cells determined by counting 300 cells per condition (*n* = 4). (C) (Left panel) Representative micrographies of EdU staining (green) and TO‐PRO‐3 (blue). Scale bar indicates 100 μm. (Right panel) Quantification of EdU‐positive cells was determined by counting 200 cells per condition (*n* = 4). (D) (Left panel) Representative western blot for LaminB1 and p21WAF1. GAPDH was used as a loading control. (Right panel) Western blot quantifications (*n* = 3). (E) (Left panel) Representative micrographies of 53BP1 staining (green) and TO‐PRO‐3 (blue). Scale bar indicates 20 μm. (Right panel) Quantification of the percentage of cells harboring more than four 53BP1 foci per nucleus determined by counting 200 cells per condition (*n* = 3). (F) Relative mRNA levels of *IL1*b, *IL8*, *CCL2*, *CXCL1*, *MMP1*, and *MMP3* were quantified by RT‐qPCR and normalized to *RPL13A*. Results are expressed as aratio related to CTL cells (*n* = 7). (G) Quantifications of the level of secreted IL8 and TGF‐β1 by ELISA assay. The level of secreted proteins is expressed as pg/mL normalized by the total number of cells per condition and is represented as IL8 and TGF‐β1 concentration per 1 million NHDFs (*n* = 3). Data information:data in (A–G) are presented as means ± SD. Statistical comparison was performed by unpaired *t*‐test. The *p*‐value shown represents difference between unexposed (CTL) and exposed (UVB) cells.

Repeated UVB exposures have been established as a robust experimental model for inducing premature senescence in various skin cell types, including dermal fibroblasts (Debacq‐Chainiaux et al. 2012) and can be considered an in vitro simplified model for dermal photoaging (Cavinato and Jansen‐Dürr 2017). Based on these findings, we generated an experimental approach in which normal human dermal fibroblasts (NHDFs) were exposed to narrowband (NB)‐UVB irradiation (500 mJ/cm^2^) twice a day for five consecutive days and confirmed that these repeated UVB doses led to stress‐induced premature senescence (UVB‐SIPS). To do so, biomarkers of senescence were all studied 3 days after the last UVB exposure. Phalloïdin staining indicated that UVB‐exposed NHDFs underwent a drastic change in morphology, adopting a star‐shaped, enlarged, and irregular morphology (Figure 2A). A minimal proportion (25%) of SA‐βgal‐positive NHDFs was observed in proliferative controls, whereas it increased to 51% in UVB‐exposed fibroblasts (Figure 2B). As expected, repeated UVB exposures led to a decrease in proliferation, as evidenced by a lower percentage of EdU‐positive cells (Figure 2C), a robust increase of p21^WAF1^, and a loss of LaminB1 (Figure 2D). Additionally, UVB‐exposed NHDFs displayed an increased proportion of nuclei with at least four 53BP1 foci (Figure 2E). Repeated UVB exposures also increased the expression of SASP‐associated genes, such as *IL1*β, *IL8, CCL2*, *CXCL1*, *MMP1*, and *MMP3* (Figure 2F), and increased IL8 and TGFβ1 secretion (Figure 2G). This confirms that UVB‐exposed NHDFs exhibit the standard hallmarks of senescence.

### 2.3 Transcriptomic Signature of UVB‐Induced Premature Senescence Revealed Involvement of Key Pathways of Senescence, Extracellular Matrix Organization, and Endoplasmic Reticulum Functions

**FIGURE 3:**
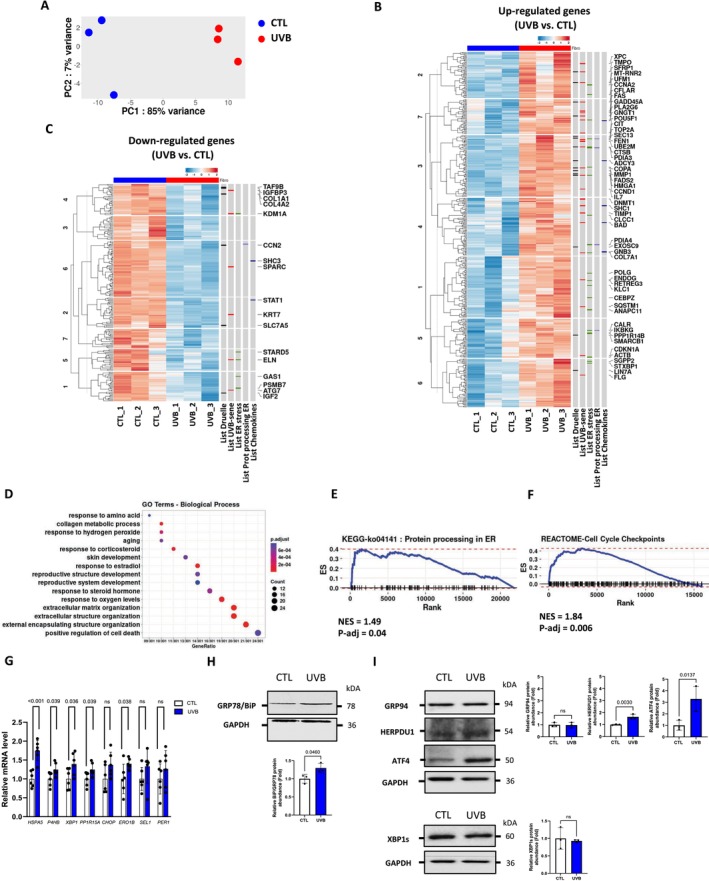
UVB‐induced premature senescent NHDFs are associated with ER stress and UPR activation. NHDFs were exposed (UVB) or not (CTL) to 500 mJ/cm^2^ UVB twice a day for 5 days. At 3 days after the last UVB exposure, mRNA and proteins were extracted. (A) Principal component analysis (PCA) of transcriptional profiles obtained via RNA‐seq for three independent experiments corresponding to the samples from unexposed (CTL) and exposed (UVB) NHDFs represented by blue or red dots, respectively. The two‐dimensional scatter plot shows the first two principal components of the analysis of all genes (*n* = 3). (B) Heatmap of the differentially upregulated genes in UVB‐induced senescent NHDFs (FDR < 0.05, log_2_(FC) ± 1.5) from UVB vs. CTL data set. (C) Heatmap of the differentially downregulated genes in UVB‐induced senescent NHDFs (FDR < 0.05, log_2_(FC) ± 1.5) from UVB vs. CTL data set. Both up‐ and downregulated gene lists were compared to gene sets published or deposited in free access databases. LIST 1: Replicative senescence signature (Druelle et al. 2016); LIST 2: GeneCards‐based ontology ≪ UVB senescence ≫; LIST 3: GeneCards‐based ontology ≪ ER stress ≫; LIST4: KEGG Protein processing in ER (ko04141); LIST 5: KEGG Chemokine Signaling Pathway (ko04062). (D) ORA (overrepresentation analysis) of biological processes (BP) gene ontology (GO) terms of differentially expressed genes in UVB vs. CTL NHDFs. Gene ratio provides the ratio of input DEGs that are annotated in BP terms. (E) GSEA enrichment plot of one of the most positively enriched pathways associated with the whole‐UVB‐induced senescent NHDFs using KEGG‐ko04141 database (NES, normalized enrichment score). (F) GSEA enrichment plot of one of the most positively enriched pathways associated with the whole‐UVB‐induced senescent NHDFs using reactome database (NES, normalized enrichment score). (G) Relative mRNA levels of *HSPA5*, *P4HB*, *XBP1*, *PP1R15A*, *CHOP*, *ERO1β*, *SEL1*, and *PER1* were quantified using RT‐qPCR and were normalized to *RPL13A*. Results are expressed as a ratio related to CTL cells (*n* = 6). (H) Representative western blots for GRP78/BiP. GAPDH was used as a loading control. Results are representative of at least three independent experiments. Western blot quantifications (*n* = 3). (I) Representative western blot for GRP94, HERPUD1, ATF4, and XBP1s. GAPDH was used as a loading control. (Right panel) Western blot quantifications (*n* = 3). Data information:Data in (G–I) are presented as means ± SD. Statistical comparison was performed using an unpaired *t*‐test. The *p*‐value shown represents difference between unexposed (CTL) and exposed (UVB) cells.

To further characterize the gene expression profile and the UVB‐induced senescence signature in NHDFs, we conducted an RNA sequencing (RNA‐seq) analysis. Differential expression analysis using principal component analysis (PCA) indicated a significant change between the transcriptional profiles of control and UVB‐induced senescent fibroblasts (UVB) (Figure 3A). Our analysis revealed that 646 genes were differentially expressed in UVB‐exposed NHDFs compared to CTL, including 430 and 216 genes that were respectively up‐ and downregulated. To identify the biological roles of the differentially expressed genes, we compared this gene list with genes belonging to one or more gene sets (Figure 3B,C). Besides, cellular functions of the differentially expressed genes were evaluated by overrepresentation analysis (ORA), a statistical method that determines whether genes from predefined sets (here, using the Gene Ontology [GO] database) are present more than would be expected (overrepresented) in our data subset. The functional enrichment analysis revealed that the differentially expressed genes in UVB‐exposed fibroblasts were most significantly enriched in biological processes related to “aging” and “skin development” (Figure 3D), in molecular function related to “growth factor binding,” and to cellular components related to “extracellular matrix” and “endoplasmic reticulum lumen” (Figure S2A,B). Other bioinformatic evaluations via gene set enrichment analysis (GSEA) using the KEGG or Reactome databases indicated that “protein processing in ER” term (Figure 3E), “cell‐cycle checkpoints” term (Figure 3F) and other cell‐cycle checkpoint gene signatures (Figure S2C) were enriched in UVB‐induced senescent fibroblasts. Finally, GSEA unveiled an almost significant enrichment of the publicly available universal senescence signature in the whole transcriptome of UVB vs. control comparison (Hernandez‐Segura et al. 2017) (Figure S2D). Based on these results and the knowledge that endoplasmic reticulum stress and UPR activation have been shown to occur in response to various senescence inducers (Denoyelle et al. 2006; Dörr et al. 2013; Blazanin et al. 2017), we chose to monitor the UPR activation status in UVB‐induced senescent fibroblasts. For that, we investigated the expression of a panel of UPR target genes downstream of ATF6α (*HSPA5*/*Grp78*, *P4HB*, *XPB1*), PERK (*CHOP*, *PP1R15A*/*GADD34*), and IRE1α (*ERO1β*, *SEL1*, *PER1*) by RT‐qPCR. Interestingly, mRNA levels of all tested genes except *CHOP*, *SEL1*, and *PER1* were significantly upregulated 3 days after the last UVB stress (Figure 3G). Moreover, immunoblotting showed that GRP78/BIP levels (but not GRP94 or XBP1s) were moderately increased in UVB‐induced senescent NHDFs, whereas ATF4 and HERPUD1 proteins were strongly accumulated (Figure 3H,I).

Collectively, these data revealed that the UVB‐induced senescence transcriptomic signature displays common senescence‐associated genes and is associated with aging, skin development, and ER stress.

### 2.4 Repeated UVB Exposures Initiate Premature Senescence in an ATF6α‐Dependent Manner

**FIGURE 4:**
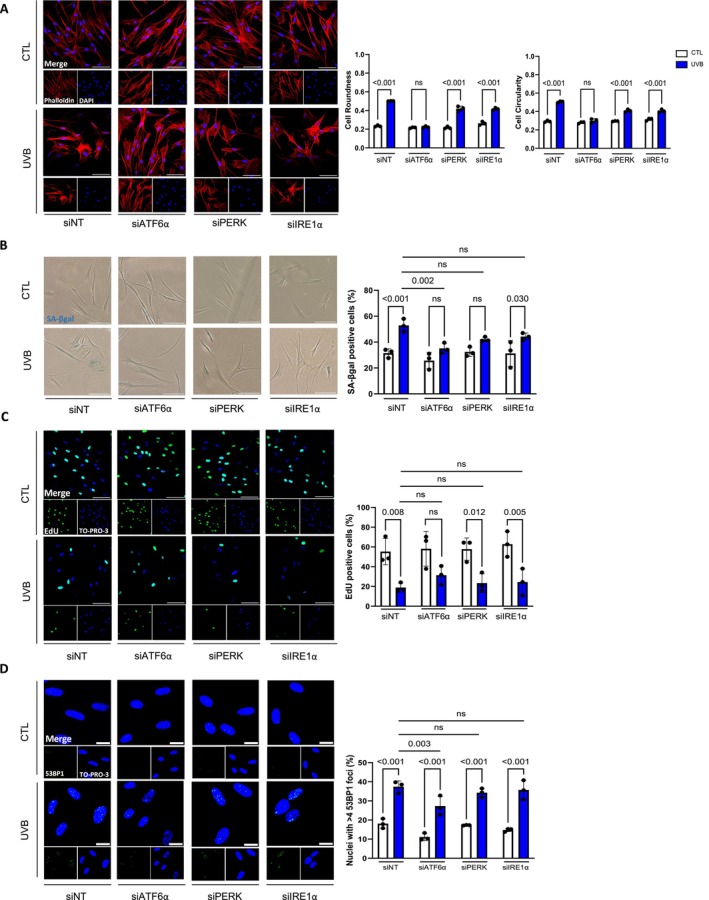
Knockdown of ATF6α prevents the complete establishment of UVB‐induced senescent phenotype. NHDFs were transfected with nontargeting control siRNA (siNT) or with siATF6α, siPERK, or siIRE1α 16 h before repetitive UVB exposures at 500 mJ/cm^2^ twice a day for five consecutive days. The biomarkers of senescence were studied 3 days after the last UVB exposure. (A) (Left panel) Representative micrographs of phalloidin (red) and DAPI (blue). Scale bar indicates 100 μm. (Right panel) Quantification of the cells circularity and the cell roundness. Circularity measures the deviation from a perfect circle (Cl = 1), whereas roundness approximates a “best‐fit” to an idealized ellipse (Ro = 1). For each condition, 50 cells have been counted (*n* = 3). (B) (Left panel) Representative micrographs of SA‐βgal staining (blue). Scale bar indicates 100 μm. (Right panel) Quantification of SA‐βgal‐positive cells determined by counting 300 cells per condition (*n* = 3). (C) (Left panel) Representative micrographies of EdU staining (green) and TO‐PRO‐3 (blue). Scale bar indicates 100 μm. (Right panel) Quantification of EdU‐positive cells was determined by counting 200 cells per condition (*n* = 3). (D) (Left panel) Representative micrographs of 53BP1 staining (green) and TO‐PRO‐3 (blue). Scale bar indicates 20 μm. (Right panel) Quantification of the percentage of cells harboring more than four 53BP1 foci was determined by counting 200 cells per condition (*n* = 3). Data information: data in (A–D) are presented as means ± SD. Statistical comparison was performed using ANOVA2 followed by Šidák's multiple comparison tests. The *p*‐value shown represents differences between unexposed (CTL) and exposed (UVB) cells and differences between nontargeting control siRNA (siNT) and siATF6α, or siPERK, and siIRE1α.

To investigate whether a functional link exists between ER stress and the onset of UVB‐induced premature senescence in NHDFs, we individually silenced the expression of each UPR sensor (ATF6α, PERK, and IRE1α) using RNA interference prior to repeated UVB exposures (Figure S3A). The knockdown efficiency of each siRNA was validated at the protein level until 8 days following siRNA transfection (Figure S3B). Transfected fibroblasts were exposed to UVB for 5 days. An RT‐PCR analysis indicated that all three sensors were upregulated 3 days after the last irradiation. The specificity of the RNA silencing was examined, demonstrating that each siRNA specifically and efficiently silenced the targeted UPR sensor still 3 days after the last UVB exposure (Figure S3C). To investigate whether silencing the expression of ATF6α, PERK, and IRE1α prior to repeated UVB exposures could impact the establishment of senescence, we first examined the major senescence hallmarks. Immunostaining of F‐actin demonstrated that upon ATF6α silencing, UVB‐exposed fibroblasts retained a spindle‐shape morphology and exhibited cell roundness and circularity similar to that of unexposed NHDFs (Figure 4A and Figure S4A). By contrast, neither PERK silencing nor IRE1α impacted the morphology of UVB‐induced senescent fibroblasts (Figure 4A). Interestingly, ATF6α and PERK (but not IRE1α) silencing prevented the increase of SA‐βgal‐positive cells after UVB exposures. However, only ATF6α silencing significantly limited the percentage of SA‐βgal‐positive NHDFs compared to UVB‐exposed fibroblasts transfected with a nontargeting control siRNA (Figure 4B). In addition, ATF6α silencing, but not IRE1α or PERK silencing, did not affect EdU incorporation in UVB‐exposed compared to control NHDFs (Figure 4C). Additionally, the increase in *p16* ^*INK4A*^ expression after UVB was no longer observed in UVB‐exposed fibroblasts knocked down for PERK, IRE1α, or ATF6α (Figure S4B). The same tendency was noted for *p21* ^*WAF1*^ (Figure S4B) suggesting that the different UPR branches may influence cell cycle regulation. Interestingly, an assessment of 53BP1 foci showed that only the silencing of ATF6α reduced the percentage of cells with at least four 53BP1 foci within the nuclei of UVB‐induced senescent NHDFs (Figure 4D). These findings suggest that ATF6α could potentially protect against the persistence of the DDR signaling.

Altogether, these data suggest that the UVB‐induced senescent phenotype is partially mediated by ATF6α. In line with this hypothesis, we investigated to what extent the addition of chemical inhibitors for each UPR branch could impair the establishment of the UVB‐induced phenotype. In this context, CA7 (Ceapin A7, ATF6α inhibitor), GSK2606414 (PERK inhibitor), or 4μ8c (IRE1α inhibitor) were added directly after the last UVB exposure (Figure S5A). The results showed similar trends, including a protective role of ATF6α inhibition using Ceapin A7 on the increased number of SA‐βgal‐positive cells and persistent DDR signaling caused by UVB exposures (Figure S5B,C). Nevertheless, these results confirm that targeting ATF6α prevents the appearance of certain biomarkers of senescence induced by UVB exposure.

### 2.5 ATF6α Silencing Impairs the Expression of Major SASP Factors After UVB Exposures

**FIGURE 5:**
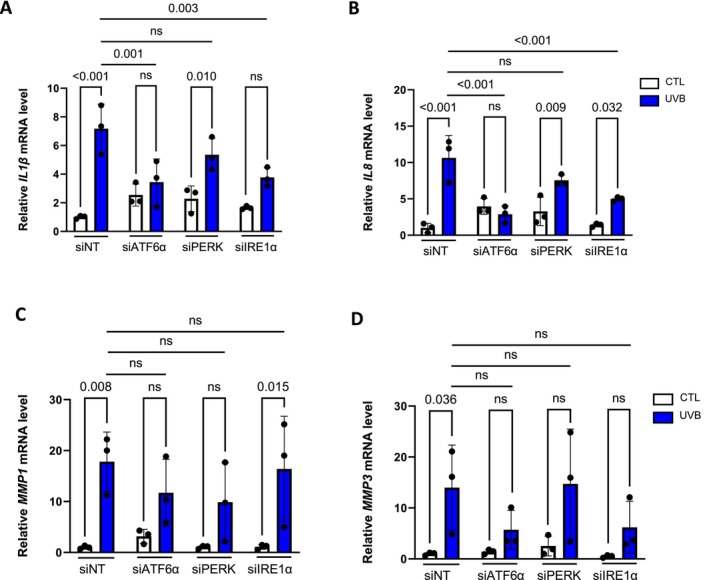
UPR disruption alters the SASP of UVB‐induced senescent fibroblasts. NHDFs were transfected with nontargeting control siRNA (siNT) or with siATF6α, siPERK, or siIRE1α 16 h before repetitive UVB exposure 500 mJ/cm^2^ twice a day for five consecutive days. Three days after the last UVB stress, mRNA was extracted. (A–D) Relative mRNA level of *IL1β* (A), *IL8* (B), *MMP1* (C), and *MMP3* (D) was quantified using RT‐qPCR and was normalized to *RPL13A*. Results are expressed as a ratio related to CTL siNT cells (*n* = 3). Data information: data in (A–D) are presented as means ± SD. Statistical comparison was performed using ANOVA2 followed by Šidák's multiple comparison tests. The *p*‐value shown represents differences between unexposed (CTL) and exposed (UVB) cells and differences between nontargeting control siRNA (siNT) and siATF6α, or siPERK, and siIRE1α.

To further investigate whether the UPR disruption could impact the establishment of UVB‐induced senescence, we analyzed the mRNA levels of genes encoding key secreted proteins associated with the Senescence‐Associated Secretory Phenotype (SASP). This includes pro‐inflammatory cytokines, as well as matrix metalloproteases (Figure 5). It is well described that the inflammatory SASP of senescent cells is mainly regulated at the transcriptomic level (Acosta et al. 2008; Kuilman et al. 2008; Rodier et al. 2009). We noted that the overexpression of *IL1β*, *IL8*, *MMP1*, and *MMP3* following repeated UVB exposures was no longer detectable upon ATF6α silencing (Figure 5A–D). It is noteworthy that IRE1α knockdown showed similar effects for *IL1β* and *MMP3*, and PERK silencing for *MMP1* (Figure 5B–D). This suggests that ATF6α silencing modifies the secretory phenotype in UVB‐induced senescence by altering the expression of major inflammatory cytokines and matrix remodeling enzymes, which can alter the tissue microenvironment.

### 2.6 Conditioned Media From UVB‐Senescent Fibroblasts Induce ATF6α‐Dependent Paracrine Keratinocyte Proliferation in RHE

**FIGURE 6:**
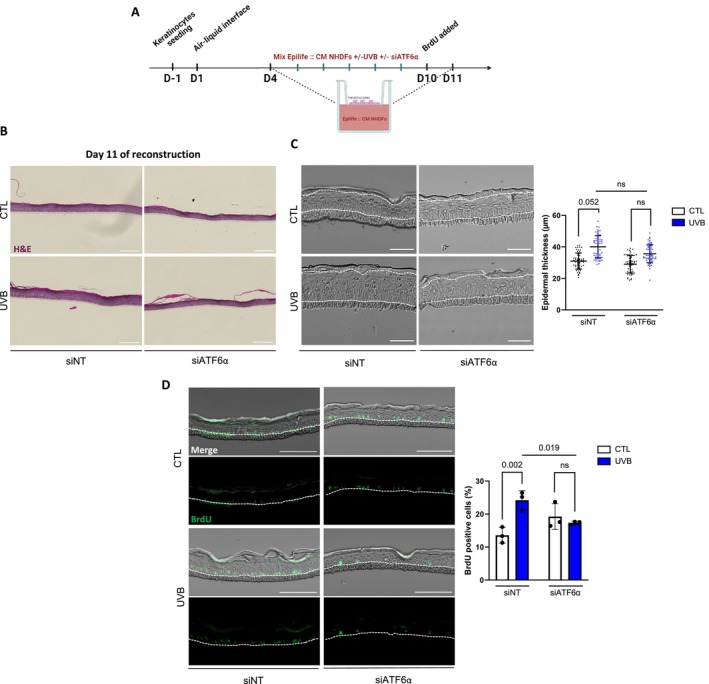
Proliferation of keratinocytes in RHEs is increased by the secretome of UVB‐induced senescent fibroblasts through an ATF6α‐dependent mechanism. Reconstructed human epidermises (RHEs) were grown using conditioned media from NHDFs exposed or not (CTL) to UVB (UVB) and transfected or not (siNT) with an siATF6α for 8 consecutive days. (A) Schematic experimental model used for the in vitro production of RHE. Epidermises were reconstructed in conditioned media derived from fibroblasts, with BrdU incorporation occurring the day before the completion of the reconstruction. (B) Representative hematoxylin–eosin (H&E) staining on histological sections prepared from RHE. Scale bar indicates 100 μm. C. (Left panel) Representative brightfield micrographies of RHE thickness. Scale bar indicates 50 μm. (Right panel) Quantification of the epidermis thickness. For each RHE, 15 measurements have been realized across the entire RHE section (*n* = 4). (D) (Left panel) Representative micrographies of BrdU staining (green) and brightfield. Scale bar represents 100 μm. (Right panel) Quantification of BrdU‐positive cells across the entire RHE section (*n* = 3). Data information: data in (C, D) are presented as means ± SD. Statistical comparison was performed by paired *t*‐test. The *p*‐value shown represents difference between unexposed (CTL) and exposed (UVB) cells, and differences between UVB siNT and UVB siATF6α.

The SASP of senescent cells can exert pleiotropic paracrine effects on the cellular environment (Birch and Gil 2020). While numerous studies have demonstrated the pro‐tumorigenic effects of SASP from senescent fibroblasts (Farsam et al. 2016; Krtolica et al. 2001; Laberge et al. 2015), few data showed its impact in a physiological context. To investigate this, we hypothesized that the alteration of SASP composition by ATF6α silencing in NHDFs could lead to changes in its impact on neighboring cells. Given that our previous RNA‐seq data associated the transcriptomic signature of UVB‐induced senescent fibroblasts with skin development, we leveraged an established model of reconstructed human epidermis (RHE) utilizing keratinocytes (Frankart et al. 2012). Additionally, we devised a novel method of reconstruction involving the contact of keratinocytes with conditioned media (CM) from fibroblasts exposed to UVB, with or without ATF6α silencing, to decipher the impact of secreted components on epidermal homeostasis. Over 11 days, keratinocytes develop into multi‐layered differentiated RHE under the influence of fibroblasts conditioned media, added from day 4 to day 11 of the reconstruction. The day before the end of the reconstruction, BrdU was added for 24 h (Figure 6A). H&E staining of sections of RHE grown in contact with CM of UVB‐induced premature senescent fibroblasts displayed the same histological structure and layer organization as those grown in contact with CM from proliferative control fibroblasts (Figure 6B). We then investigated the impact of CM from UVB‐exposed fibroblasts silenced for ATF6α on keratinocyte differentiation. The layer organization of keratinocytes exposed to control CM or to CM from senescent fibroblasts, whether silenced for ATF6α or not, appeared similar (Figure S6). Immunofluorescence staining of early (K10) and late (Involucrin, Loricrin) differentiation markers revealed no differences, indicating that the differentiation process is not impaired by the CM (Frankart et al. 2012) (Figure S6A–C). However, CM from UVB‐exposed fibroblasts appeared to significantly increase the epidermal thickness of RHE, and surprisingly, this effect was no longer observable in RHE grown with CM from fibroblasts silenced for ATF6α (Figure 6C). In accordance, further analysis revealed that UVB‐conditioned media significantly increased the percentage of proliferative keratinocytes from 13.5% to 24%, and the knockdown of ATF6α was sufficient to completely prevent this effect (Figure 6D). These findings provide the first evidence that some secreted components from UVB‐induced senescent fibroblasts' secretome can induce keratinocyte hyperproliferation and epidermal thickening in an ATF6α‐dependent manner.

### 2.7 Increased Proliferation of Keratinocytes Is Mediated by an ATF6α/IL8 Axis

**FIGURE 7:**
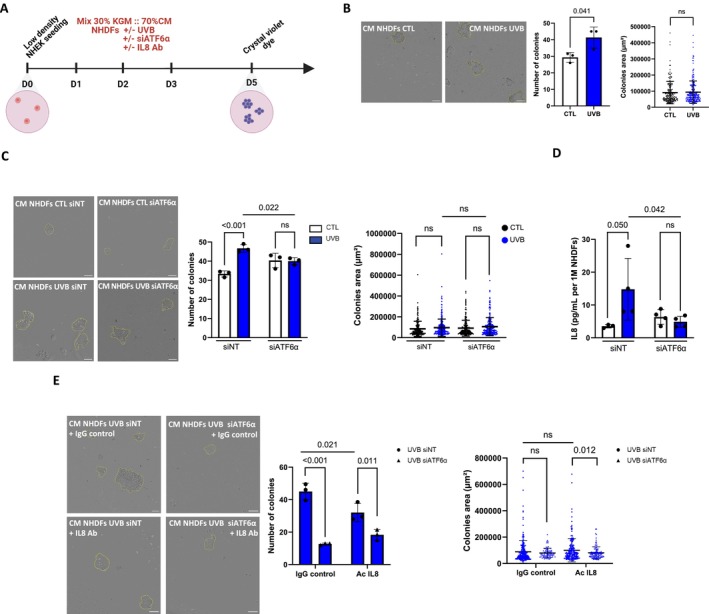
Inhibition of secreted IL8 replicates ATF6α invalidation effect on keratinocyte proliferation. NHEKs were grown in 24‐well plates using conditioned media from NHDFs exposed to UVB (UVB), invalidated or not (siNT) for ATF6α (siATF6α) for five consecutive days, and supplemented or not with an IL‐8‐neutralizing antibody. (A) Schematic experimental model used to evaluate keratinocyte proliferation. NHEKs were seeded at a low density and then cultured in a mixed media, containing 70% conditioned media derived from fibroblasts. The media were changed every day for 3 days, and the ability of keratinocytes to form colonies was evaluated after staining with 0.025% crystal violet. Then, gray scale pictures of each well were acquired using Incucyte SX3. (B, C) (Left panel) Representative pictures of NHEKs forming colonies at day 5. Scale bar represents 200 μm. (Right panel) Quantification of colonies formed and colonies area after 5 days (*n* = 3). (D) Quantification of the level of secreted IL8 in UVB‐induced senescent fibroblasts silenced or not (siNT) for ATF6α (siATF6α) by ELISA assay. The level of secreted proteins is expressed as pg/mL normalized by the total number of cells per conditions and is represented as IL8 concentration per 1 million NHDFs (*n* = 3–4). (E) An IL8‐neutralizing Ab or a control IgG was added to the conditioned media from UVB‐induced fibroblasts transfected with nontargeting control siRNA (siNT) or siATF6a. (Left panel) Representative pictures of NHEKs‐forming colonies at day 5. The scale bar represents 200 μm. (Right panel) Quantification of colonies formed and colonies area after 5 days (*n* = 3). Data information: Data in (B–E) are presented as means ± SD. Statistical comparison was performed by unpaired *t*‐test. *p*‐value shown represents difference between CTL and UVB, or UVB siNT and UVB siATF6α, or UVB siNT IgG control and UVB siNT IL8 Ab, or UVB siATF6α IgG control and UVB siATF6α IL8 Ab.

To further describe the pro‐proliferative effect of CM from UVB‐induced fibroblasts on keratinocytes, we took advantage of the ability of keratinocytes to form colonies and conducted a clonogenic assay to assess their proliferation (Rafehi et al. 2011). To achieve this, keratinocytes were seeded at low density and incubated with conditioned media from NHDFs exposed or not to UVB and invalidated or not for ATF6α for 3 days. Colonies were fixed 48 h after the last media change, and only colonies with at least 20 cells were counted (Figure 7A). We observed a significant increase in the number of colonies when keratinocytes were grown with CM from UVB‐exposed fibroblasts compared to CM from proliferative control fibroblasts, whereas no differences regarding colony area were observed (Figure 7B). Interestingly, focusing on CM from UVB‐exposed fibroblasts, we showed that ATF6α invalidation allowed us to significantly reduce the number of colonies formed, without impacting colony area (Figure 7C). To go further into our understanding of ATF6α‐dependent effectors involved in keratinocyte proliferation, we took advantage of our findings concerning the expression of SASP factors as depicted in Figure 5. Among the components under the control of ATF6α, and based on the literature, we identified IL8 as a potent candidate. Although little is known about the biological effects of IL8 on epidermal keratinocytes, a study showed that the addition of exogenous IL8 in a culture medium stimulates keratinocyte proliferation (Steude et al. 2002). To delve deeper into this hypothesis, the secreted level of IL8 was quantified by ELISA, revealing that ATF6α invalidation significantly reduces the quantity of IL8 in UVB‐induced senescent fibroblasts from 14.8 to 4.9 pg/mL/1 million fibroblasts (Figure 7D). To determine whether IL8 was necessary for the hyperproliferation of keratinocytes induced by CM from UVB‐exposed fibroblasts, a neutralizing antibody against IL8 was freshly added to the CM daily. Results showed a significant decrease in colony formation with IL8 inhibition accompanied by a weak decrease in colony area (Figure 7E), supporting our hypothesis regarding the paracrine effects of UVB‐induced senescent fibroblasts on keratinocyte proliferation, possibly mediated by an ATF6α/IL8 axis. This demonstrates that ATF6α can control IL8 production and promote epidermal hypertrophy by impacting keratinocyte proliferation.

## 3 Discussion

To date, studies have established the involvement of UPR in various models of senescence, with specific branches being activated depending on the cell type and the senescence inducer (reviewed in; Abbadie and Pluquet 2020). We first highlighted in ex vivo human skin samples that UPR‐related proteins are overexpressed in dermal (photo)aging. More specifically, through a combination of in vitro studies on skin cells and an experimental model of reconstructed human epidermis, we have uncovered the regulatory role of the ATF6α branch of the UPR in the onset of UVB‐induced senescence and highlighted its potential effect in maintaining epidermal homeostasis under stress. The silencing of ATF6α prevented the major hallmarks of the UVB‐induced senescent phenotype in NHDFs, evidenced by the prevention of increased SA‐βgal positive cells and morphological changes, and selective alteration of the SASP. Interestingly, several studies have shown that the SASP can spread to neighboring normal cells and impair their function (reviewed in Gonzalez‐Meljem et al. 2018). However, these studies often focused on the effect of senescent cells in the tumor microenvironment, promoting the growth and proliferation of tumor cells, but did not address the intercommunication of senescent cells in a physiological context. Here, we identified an unexpected consequence of conditioned media from UVB‐induced senescent fibroblasts in causing keratinocyte hyperproliferation in an ATF6α/IL8‐dependent mechanism. However, we cannot exclude the potential participation of other SASP factors regulated by ATF6α in these processes, which remain to be investigated.

In vitro, cell exposure to UV radiation has been linked to the activation of adaptive stress responses, with cellular senescence being among the most extensively studied (Moon et al. 2019). Additionally, chronic UV exposure can induce other biological responses such as endoplasmic reticulum (ER) stress and activation of the unfolded protein response (UPR) (Komori et al. 2012; Karagöz et al. 2019). However, the in vivo contribution of the UPR in skin aging remains poorly studied. We found that UPR markers are detected in the dermis and increase with age. Similar results were observed in aged sun‐exposed dermis compared to young sun‐exposed counterparts. This indicates that dermal fibroblasts elicit UPR during skin aging, but this seems to be tissue‐specific since a decrease in UPR activation has also been reported in other models of aging (Chalil et al. 2015). This observation prompted us to investigate the role of UPR branches in UVB‐induced senescence in dermal fibroblasts and the molecular mechanisms contributing to age‐related changes in the skin.

Previous studies have demonstrated that UV‐induced senescent human dermal fibroblasts display several common biomarkers of senescence (Berneburg et al. 2004; Herrmann et al. 1998; Debacq‐Chainiaux et al. 2005). In our study, we used normal human dermal fibroblasts, necessitating adjustments in the UVB dose (doubled) compared to the original model related to normal human fetal dermal fibroblasts. This adaptation enabled the induction of a senescent phenotype, characterized by SA‐βgal positive cells, enlarged morphology, cell cycle arrest, persistent DNA damage, and establishment of the SASP in NHDFs, which were consistent with the literature (Saul et al. 2022). Interestingly, ER stress is now admitted as one of the signaling pathways connected to senescence (reviewed in; Pluquet et al. 2015; Hernandez‐Segura et al. 2018). Indeed, we and others have shown the involvement of ER stress and UPR activation in replicative and OIS‐induced senescence models (Druelle et al. 2016; Drullion et al. 2018; Blazanin et al. 2017). In this study, we clearly showed that UVB‐induced senescent fibroblasts are associated with ER stress and UPR activation. Additionally, we demonstrated that silencing the UPR before repeated exposures to UVB altered the complete establishment of the senescent phenotype. We found that ATF6α silencing, more than PERK and IRE1α silencing, can prevent the establishment of most UVB‐induced senescent biomarkers. This observation reinforces the suggested role of ATF6α as a key player in various types of senescence (Druelle et al. 2016; Kim et al. 2019). However, the importance of PERK and IRE1α should not be overlooked, as we observed that silencing of PERK prevents the increased proportion of SA‐βgal‐positive cells, while silencing of IRE1α prevents the overexpression of *IL1β* and *MMP3*. In comparison, we showed that ATF6α silencing impacts SA‐βgal, morphology, SASP composition, and may influence persistent DDR signaling.

Interestingly, it has been recently demonstrated in cancer cells that ATF6α can prevent DNA damage in response to the cytotoxic effects of ER stressors (Benedetti et al. 2022). This effect involves the stabilization of BRCA‐1 through the activation of mTOR signaling by ATF6α. Several groups have already reported a link between ATF6α and activation of mTOR signaling in different biological contexts (Blackwood et al. 2019; Allen and Seo 2018; Schewe and Aguirre‐Ghiso 2008), and others have described mTOR as necessary for SASP control and particularly for IL6 and IL8 expression (Laberge et al. 2015; Herranz et al. 2015). These results supported the possibility that in our model, the ATF6α‐dependent SASP composition may involve mTOR. However, we cannot rule out the implication of other pathways involved in the regulation of inflammatory SASP factors, such as the DDR or cGAS/STING, as none of these hypotheses have been investigated in this study (Bolland et al. 2021; Rösing et al. 2024).

To study the functional consequences of paracrine factors regulated by ATF6α, we used reconstructed human epidermis composed of normal human keratinocytes to partly mimic the human epidermal environment (Frankart et al. 2012). We observed that conditioned media from UVB‐induced fibroblasts thickened the epidermis without altering the expression of differentiation markers in RHE. Interestingly, this thickening resembles the phenotype induced directly by keratinocyte injury after chronic UVB exposure (Scott et al. 2012) or acute UVB exposure (Koehler et al. 2015), suggesting that paracrine factors released by UVB‐induced fibroblasts may partially overlap some effects of direct UVB exposure on keratinocytes. Surprisingly, we highlighted that this process involves paracrine factors under the control of ATF6α. We identified IL8 as a factor influencing keratinocyte proliferation. Remarkably, IL8‐neutralizing antibodies added to conditioned media from UVB‐induced fibroblasts were able to prevent the increased number of keratinocyte clones. This experiment revealed that IL8 selectively promotes keratinocyte proliferation, in addition to its known chemotactic properties. Furthermore, the addition of exogenous IL8 in an organotypic culture of keratinocytes has been shown to result in marked hyperproliferation (Steude et al. 2002). Thus, these results connect the ATF6α/IL8 axis to pro‐proliferative phenotypes, shedding light on ATF6α's role as a potential regulator of a key SASP component. Nevertheless, the mechanistically regulatory relationship between ATF6α and IL8 remains unclear.

Besides, Saul et al. reported that distinct subpopulations of senescent cells may coexist in vivo, including a p21^CIP1^‐only positive cell population, a p16^INK4^‐only positive cell population, and a population of cells co‐expressing p21^CIP1^/p16^INK4^, with distinct SASP compositions. Although it remains unknown if the first category switches toward the second category (Saul et al. 2022), this would lead to the idea that the senescent phenotype evolves over time (Wang et al. 2024), impacting the transcriptome of senescent cells (Hernandez‐Segura et al. 2017) as well as their SASP composition in vivo (Wang et al. 2024). In our study, the specific senescence time point we analyzed (3 days after the last UVB exposure) corresponds to the time required for the senescent phenotype to be fully established after repeated UVB exposures, limiting the temporal dynamics of the senescent phenotype. Additionally, the SASP is known to be dependent on the senescence stimuli and the cell type (Basisty et al. 2020; Ito et al. 2017) and varies over time (Schmitt 2016). Therefore, SASP can modulate the fate of normal neighboring cells in several ways. For example, the early secretion of PDGF‐AA SASP factor by senescent cells affects myofibroblast differentiation (Demaria et al. 2014), whereas long‐term exposure of primary keratinocytes to SASP from senescent cells promotes senescence and reduces regenerative capacities (Ritschka et al. 2017). This aligns well with our findings, suggesting that the transient presence of SASP from senescent fibroblasts may initially stimulate processes such as keratinocyte proliferation before potentially leading to the establishment of a chronic inflammatory environment. Indeed, several skin‐related diseases have been linked to keratinocyte hyperproliferation, such as psoriasis, atopic dermatitis (Niehues et al. 2022), and in severe cases, actinic keratoses (AK). Actinic keratoses are precancerous skin lesions arising from the excessive proliferation of transformed neoplastic keratinocytes in the epidermis, caused by solar UV radiation. Common among the elderly population, actinic keratoses present an elevated risk of developing into squamous cell carcinoma (SCC) (Berman et al. 2013). In our model, normal keratinocytes were not directly exposed to UVB but were grown with conditioned media from UVB‐induced senescent fibroblasts. This suggests that, aside from direct acute physical stressors, long‐term alterations in the cellular microenvironment could also have additional effects on tissue homeostasis. Nevertheless, since the skin is a more complex organ than the simplified 3D model used here, the effects of fibroblast secretions on keratinocytes require further in vivo validation, accounting for the multidirectional interactions between all skin‐resident cells.

Targeting senescent cells holds great promise. Emerging senotherapeutic strategies fall into two categories: senolytics, which specifically kill senescent cells, and senomorphics, which suppress certain properties of senescent cells, primarily SASP factors. Several studies reported senomorphic properties of plant extracts exerting senomorphic properties on senescent dermal fibroblasts, epidermal keratinocytes, and melanocytes (reviewed in Csekes and Račková 2021; Giroud et al. 2023). Most of the time, these studies only assessed a few major SASP factors following senomorphic treatments. Nevertheless, it has been shown that the secretome from senescent preadipocytes treated with ruxolitinib (a JAK inhibitor) prevents macrophage migration, reduces inflammation in nonsenescent preadipocytes (Xu et al. 2015), and attenuates osteoclast differentiation (Farr et al. 2017). Moreover, the secretome from simvastatin‐treated senescent fibroblasts suppressed breast cancer cell proliferation (Liu et al. 2015). Further experiments are needed to explore whether blocking the SASP with senomorphics leads to a less deleterious SASP that could be considered modified rather than nonsenescent‐like, or whether it may increase the secretion of some detrimental factors.

Nevertheless, very few studies have investigated the senotherapeutic approach in skin aging and age‐related disorders. Azameh et al. showed that a prolonged expression of p16^INK4A^ in mouse epidermis leads to hyperplasia and dysplasia and that senolytic elimination of p16‐positive cells suppresses hyperplasia (Azazmeh et al. 2020). In this respect, further experiments are required to determine whether senotherapy could revert skin aging. In conclusion, this work has identified the ATF6α/IL8 axis in UVB‐induced senescent fibroblasts as a possible mechanism controlling paracrine proliferation in surrounding normal keratinocytes. Understanding the regulation of SASP and developing strategies to counteract its effects on skin aging and age‐related skin disorders is imperative for future research. Finally, manipulating the ATF6α pathway may offer potential therapeutic opportunities to target senescent cells by modulating SASP composition.

## 4 Materials and Methods

### 4.1 Immunofluorescence on Human Dermis Samples

Human skin samples were obtained from the SkinAge Project (SKINAGE: NCT02553954) at Oscar Lambret Centre (Lille, France), Department of Anatomopathology, in compliance with French regulations. Skin punch biopsies of 5 mm were collected from healthy volunteers, including young donors aged 19–39 years, with 3 males for sun‐exposed samples and 2 males and 1 female for nonsun‐exposed samples, as well as older donors aged 58–82 years, with 1 male and 2 females for sun‐exposed samples and 2 males and 1 female for nonsun‐exposed. The study was approved by local (Le Comité de Protection des Personnes Nord‐Ouest) and national (Agence nationale de sécurité du médicament et des produits de santé) ethics committees. Participants provided informed written consent prior to inclusion in this study. Biopsies were embedded at optimal‐cutting temperature into plastic cryomolds before freezing. After frozen sectioning (6‐μm‐thick) on a microtome‐cryostat, sections were mounted onto slides for analysis. Sections were fixed in paraformaldehyde 4% for 10 min and washed in PBS. Nonspecific binding was blocked by incubation in 5% BSA/PBS. The primary antibody was incubated overnight at 4°C. The used antibodies were anti‐vimentin (AF2105, 1:100, R&D Systems, Minneapolis, MN, USA), anti‐XBP1s (2G4‐3 E11‐3 E9, 1:50) kindly provided by Eric Chevet (Inserm U1242, University of Rennes, France), anti‐PDI (Ab2792, 1:100; Abcam, Cambridge, UK), anti‐HERPUD1 (H00009709‐M04, 1:300; Abnova, Taipei, Taiwan), anti‐53BP1 (NB100‐304, 1:400; Novus Biologicals, Englewood, CO, USA), anti‐LaminB1 (Ab16048, 1:500; Abcam), or anti‐GRP78 (sc‐1050, 1:100; Santa Cruz Biotechnology, Dallas, TX, USA). After washes in PBS, tissue sections were incubated with secondary goat anti‐mouse IgG antibody Alexa Fluor 555 (A21422, 1:1000; Invitrogen, Waltham, MA, USA), donkey anti‐rabbit IgG antibody Alexa Fluor 568 (A10042, 1:1000; Invitrogen), or donkey anti‐goat IgG antibody Alexa Fluor 488 (A11055, 1:1000; Invitrogen) for 1 h at room temperature. For double immunofluorescence, the two primary and two secondary antibodies were co‐incubated. Finally, tissue sections were washed in PBS, and nuclei were stained for 5 min with DAPI (D9542; Sigma‐Aldrich, St Louis, MO, USA) at 2 μg/mL and mounted using Dako Glycergel Mounting Medium (C0563; Dako, Santa Clara, CA, USA). Images were acquired on a confocal Zeiss (LSM880; Airyscan, Oberkochen, Germany).

### 4.2 Isolation of Primary Dermal Fibroblasts

Normal human dermal fibroblasts (NHDFs) were isolated from the foreskins of young and healthy donors (aged < 10 years) as previously described (Tigges et al. 2013). Skin samples were obtained from Clinique St‐Luc (Dr. L. de Visscher, Bouge, Belgium) following the approval of the Medical Ethical Committee of Clinique St‐Luc and according to the principles set out in the Declaration of Helsinki.

### 4.3 Cell Cultures

NHDFs and normal human fetal dermal fibroblasts AG04431 (Coriell Institute for Medical Research) were cultured in Basal Medium Eagle (BME, 41010026; Gibco) supplemented with 10% fetal bovine serum (FBS; Corning), 2 mM L‐glutamine (Thermo Fisher Scientific, Waltham, MA, USA), and penicillin–streptomycin (50 U/mL).

Human Epidermal Keratinocytes neonatal, HEKn (Thermo Fisher Scientific, C0015C) used for reconstructed human epidermises, were grown in a specific keratinocyte serum‐free medium, completed with bovine pituitary extract, epidermal growth factor, insulin, hydrocortisone, transferrin, calcium, penicillin, and streptomycin (KGM‐2 BulletKit, CC‐3107, Lonza, Basel, Switzerland).

Normal human epidermal keratinocytes (NHEKs, 00192627; Lonza) used for clonogenic assays were grown in KGM‐Gold Keratinocyte Basal Medium (KGM‐Gold Keratinocyte Basal Medium BulletKit, 192,060, containing basal medium and supplements).

### 4.4 UVB‐Induced Senescence, RNA Interference, and UPR Inhibition

NHDFs at their exponential growth phase were seeded at 4000 cells/cm^2^ (CTL) or 8000 cells/cm^2^ (UVB) (or at 8000 cells/cm^2^ for AG004431) in BME with 1% FBS, 2 mM L‐glutamine, and penicillin–streptomycin (50 U/mL). At day three after seeding, cells were exposed to NB‐UVB (peaking at 312 nm) (TL20W/01; Philips) at 500 mJ/cm^2^ two times a day for 5 days (or at 250 mJ/cm^2^ for AG004431 cells, as previously described in Debacq‐Chainiaux et al. (2005)).

For ATF6α, PERK, or IRE1α knockdown, NHDFs were transfected 48 h after seeding with specific siGENOME SMARTpool (Dharmacon, Lafayette, CO), siATF6α (GAACAGGGCUCAAAUUCUC, AACCAAAUCUGUACAGUUA, UCACACAGCUCCCUAAUCA, GAACAGGAUUCCAGGAGAA), siPERK (CCAAGAUGCUGGAGAGAUU, GGAAGUACCAGCACAGUGA, AGAACAAGCUCAACUACUU, CCCAAAAGCCUUACGGUCA), or siIRE1α (CCAAGAUGCUGGAGAGAUU, GGAAGUACCAGCACAGUGA, AGAACAAGCUCAACUACUU, CCCAAAAGCCUUACGGUCA) at 25 nM using DharmaFECT 1 (Dharmacon) transfection reagent according to the manufacturer's instructions. A siGENOME nontargeting control siRNA pool (D‐001206‐13, Dharmacon), siNT (UAAGGCUAUGAAGAGAUAC, AUGUAUUGGCCUGUAUUAG, AUGAACGUGAAUUGCUCAA, UGGUUUACAUGUCGACUAA) was used as a control. The following day, NHDFs were exposed to UVB as described above.

For ATF6α, PERK, or IRE1α inhibition, AG04431 cells were treated directly after the last UVB exposure for up to 72 h with, respectively, Ceapin‐A7 (CA7, 10 μM, SML2330, Sigma‐Aldrich), GSK2606414 (GSK, 10 μM, 516535, Sigma‐Aldrich), 4μ8c (4μ8c, 100 μM, SML0949, Sigma‐Aldrich), or with DMSO (A994.1, Carl Roth, Karlsruhe, Germany) as a control.

### 4.5 SA‐βgal Staining

Three days after the last UVB exposure, cells were detached and seeded at a density of 15,000 cells per well in technical triplicates within six‐well plates. The next day, cytochemical detection of SA‐βgal activity was carried out as previously described (Debacq‐Chainiaux et al. 2009).

### 4.6 EdU Staining

Three days after the last UVB exposure, cells were detached and seeded onto glass coverslips. The next day, cells were incubated with 10 μM EdU for 16 h before being fixed with 4% paraformaldehyde (Merck, Rahway, NJ). EdU detection was performed using the Click‐iT EdU Cell Proliferation Kit (BCK‐EDU488, Sigma‐Aldrich) according to the manufacturer's instructions. Cell nuclei were counterstained with TO‐PRO‐3 (T3605, 1:80 in RNAse 2 mg/mL, Thermo Fisher Scientific). Images were acquired on Leica Microsystems (TCS SP5; Leica, Wetzlar, Germany).

### 4.7 Immunofluorescence of Cultured Cells

Three days after the last UVB exposure, cells were detached and seeded onto glass coverslips. The next day, cells were fixed with 4% paraformaldehyde (Merck) and permeabilized using 1% Triton X‐100 in PBS (Sigma‐Aldrich).

For the detection of 53BP1, the primary antibody anti‐53BP1 (N100‐305, 1:2000, Novus) was incubated overnight at 4°C using 3% BSA in PBS, followed by the secondary goat anti‐rabbit IgG antibody Alexa Fluor 488 (A11008, Invitrogen) for 1 h at room temperature; then nuclei were counterstained with TO‐PRO‐3 (T3605, 1:80 in PBS‐RNAse 2 mg/mL, Thermo Fisher Scientific).

For the detection of actin, the phalloidin probe (A12380, 1:100, Thermo Fisher Scientific) was incubated overnight at 4°C using 3% BSA in PBS, followed by nuclei counterstaining with DAPI (D9542, in PBS 1 μg/mL, Sigma Aldrich). Images were acquired with a Broadband Confocal TCS SP5 (Leica).

### 4.8 RNA Isolation and RT‐qPCR

Total RNA was isolated from cultured cells using the ReliaPrep RNA Tissue Miniprep System (Z6111, Promega) and reverse transcribed using the GoScript Reverse Transcriptase Kit (A2791, Promega) in a final volume of 20 μL following the manufacturer's instructions. Semi‐quantitative real‐time polymerase chain reaction (PCR) was performed using GoTaq qPCR Mix (Promega), primers (sequences are provided in Table 2), and the Viaa7 instrument (Applied Biosystems). Relative mRNA level was determined using the ∆∆Ct method normalized to the mRNA abundance of *RPL13A* and expressed relative to the stated control (Schmittgen and Livak 2008).

**TABLE 2:** List of primers used.

Target	Forward	Reverse
*p16*	GCCCAACGCACCGAATAGT	CGCTGCCCATCATCATGAC
*p21*	CTGGAGACTCTCAGGGTCGAA	CCAGGACTGCAGGCTTCCT
*HSPA5*	GGTGTGCTCTCTGGTGATCAAG	ATGACACCTCCCACAGTTTCAAT
*P4HB*	AGTGACGTGTTCTCCAAATACC	AAAGTTGTTCCGGCCTTCAT
*XBP1*	TGCCAGAGATCGAAAGAAGG	CCTGGTTCTCAACTACAAGGC
*PP1R15A*	GGACACTGCAAGGTTCTGAT	CCGGTGTGATGGTGGATAAG
*CHOP*	GCAAGAGGTCCTGTCTTCAGATG	CTCAGTCAGCCAAGCCAGAGA
*ERO1B*	GAGCCCTGTCCAGAGAGTAA	GCTCCCAGTTTATTAGCTTGC
*SEL1*	GCAGCGAGAGAGATGTTTGA	CACCAAGTCCAGAGGCATAC
*PER1*	GCGTGCGGAGGACACT	GAGGGAGTGAGGTGGAAGAT
*ATF6α*	GATGATGAAAAATGGAGCAGCTT	AGACTGAAGAGCAGGTGAGCAAA
*PERK*	ATGCTTTCACGGTCTTGGTC	TCATCCAGCCTTAGCAAACC
*IRE1α*	CGAAACTTCCTTTTACCATCCC	CGATGACAAAGTCTGCTGCTT
*IL1β*	GCCCTAAACAGATGAAGTGCTC	GAGATTCGTAGCTGGATGCC
*IL8*	CTGGCCGTGGCTCTCTTG	GGGTGGAAAGGTTTGGAGTATG
*MMP1*	TCGGGGAGAAGTGATGTTCT	GTCGGCAAATTCGTAAGCAG
*MMP3*	CCCTTTTGATGGACCTGGAA	CATGAGCAGCAACGAGAAATAA
*RPL13A*	GCCTACAAGAAAGTTTGCCTATCTG	TGAGCTGTTTCTTCTTCCGGTAGT

### 4.9 Protein Extraction and Western Blot

Total cell protein extracts were harvested using homemade lysis buffer (40 mM Tris; pH 7.5, 150 mM KCl, 1 mM EDTA, and 1% Triton X‐100) supplemented with a protease inhibitor cocktail (Roche). Protein extracts were then incubated on a rotating wheel for 30 min at 4°C before centrifugation for 10 min at 16,000 *g*, at 4°C, to collect supernatants. Protein concentration was determined with Pierce 660 nm Protein Assay Reagent (22,660, Thermo Fisher Scientific), complemented with Ionic Detergent Compatibility Reagent (IDC) according to the manufacturer's recommendations. For GRP78/BiP, PERK, and IRE1α blots, equal protein concentration (5–10 μg) was loaded onto SDS‐PAGE homemade polyacrylamide gels and then transferred to PolyVinyliDene Fluoride (PVDF) membrane (Immobilon‐P, Merck Millipore, Burlington, MA, USA). Membranes were blocked for 1 h at room temperature in blocking solution Intercept Blocking Buffer (TBS‐T, LI‐COR Biosciences, Lincoln, NE, USA) and then incubated with primary antibodies overnight at 4°C diluted in TBS supplemented with 0.1% Tween‐20 (Carl Roth). The used antibodies were anti‐PERK (3192, 1:1000, Cell Signaling, Danvers, MA, USA), anti‐IRE1α (3294, 1:1000, Cell signaling), anti‐GRP78 (3177, 1:1000, Cell Signaling), anti‐GAPDH (G8795, 1:1000, Sigma‐Aldrich or Ab128915, 1:1000, Abcam). After washes in homemade TBS, membranes were incubated with secondary antibody goat anti‐rabbit IRDye 800CW (926‐322,111, LI‐COR Biosciences) or with goat‐anti‐mouse IRDye 680RD (926–68070, LI‐COR Biosciences) 1 h at room temperature in TBS‐T supplemented with 0.1% Tween‐20 (9127.1, Carl Roth) and 0.01% SDS (A3942, Carl Roth). Membranes were finally dried for 1 h at 37°C and scanned using Amersham Typhoon.

For ATF6α, GRP94, XBP1s, HERPUD1, ATF4, LaminB1, and p21 blots, equal protein concentration (4–5 μg) of proteins was loaded onto SDS‐PAGE homemade polyacrylamide gels and then transferred to nitrocellulose membrane (88,018, Thermo Fisher Scientific). The membrane was blocked for 90 min using 5% nonfat milk in PBS and then incubated with anti‐ATF6α (Ab122897, 1:1000, Abcam), or anti‐GRP94 (NB300‐619, 1:200, Novus), or anti‐XBP1s (1:1500) kindly provided by Eric Chevet (Inserm U1242, University of Rennes, France), or anti‐HERPUD1 (H00009709‐M04, 1:500, Novus), or anti‐ATF4 (sc‐200, 1:200, Sant Cruz Biotechnology), or anti‐LaminB1 (Ab16048, 1:10,000, Abcam), or anti‐p21 (2947S, 1:2000, Cell Signaling) overnight at 4°C in the blocking solution. After washes in PBS supplemented with 0.1% Tween‐20 (Carl Roth), the membranes were incubated with secondary antibody, goat anti‐mouse coupled with HRP (115–035‐146, 1:10,000, Jackson ImmunoResearch, West Grove, PA, USA), or goat anti‐rat coupled with HRP (112‐035‐167, 1:10,000, Jackson ImmunoResearch), or goat anti‐mouse coupled with HRP (115‐035‐146, 1:10,000, Jackson ImmunoResearch), or donkey anti‐rabbit coupled with HRP (711‐035‐152, 1:10,000, Jackson ImmunoResearch) for 45 min at room temperature in the blocking solution. Blots were revealed using the SuperSignal West Femto Maximum Sensitivity Substrate (34,096, Thermo Fisher Scientific) and scanned using Bio‐Rad ChemiDoc XRS+ (Bio‐Rad, Hercules, CA, USA) except for GRP94 that was revealed using the Amersham ECL Western Blotting Detection Reagent (RPN2209, Cytiva, Marlborough, MA, USA). For ATF6α blot only, GAPDH was used as a loading control (Ab128915, 1:1000, Abcam) and revealed using Amersham Typhoon. For other blots, GAPDH (sc‐32233, 1:200, Sant Cruz Biotechnology) was used and revealed using Bio‐Rad ChemiDoc XRS+.

### 4.10 Conditioned Media (CM)

At 48 h after the last UVB exposure, NHDFs were washed three times with PBS, and the medium was changed to serum‐free medium. Cells were cultured for an additional 16 h, and conditioned media were centrifuged at 200 *g* at 4°C for 5 min to remove cellular debris, and the media supernatant was filtered using a 0.2 μm filter. Conditioned media were then aliquoted and frozen at −20°C until use.

### 4.11 Reconstructed Human Epidermis (RHE)

RHE was produced following the protocol described by Frankart et al. (2012). Briefly, HEKs at early passage were thawed in a complete KGM‐2 medium. The following day, the medium was replaced by a complete Epilife medium (Gibco, MEPI500CA). When keratinocytes reached 70%–80% of confluency, HEKs were seeded on a polycarbonate filter (Millipore, PIHP01250) in complete EpiLife medium supplemented with 1.5 mM Ca^2+^, at a density of 250,000 cells/cm^2^. The next day, keratinocytes were exposed to the air–liquid interface for 11 days, and the medium under the polycarbonate filter was replaced by complete EpiLife medium supplemented with 1.5 mM Ca^2+^, 10 ng/mL keratinocyte growth factors (KGF, R&D systems), and 50 μg/mL vitamin C.

At 4 days after exposure to the air–liquid interface, the complete Epilife medium under the polycarbonate filter was mixed with CM from NHDFs exposed or not (CTL) to UVB and transfected with siNT or siATF6α. CM was normalized to 95,000 cells/mL using serum‐free BME and then diluted to a 50%–50% ratio with complete Epilife medium supplemented with 1.5 mM Ca^2+^, 10 ng/mL keratinocyte growth factors (KGF, R&D systems), and 50 μg/mL vitamin C. The medium was changed every 2 days. At day 10, bromodeoxyuridine (BrdU, 10 μM, b9285, Sigma) was added to the mixed medium. The reconstruction was stopped on day 11.

RHE was fixed after 11 days of reconstruction in 4% formaldehyde for at least 24 h. The following day, they were dehydrated in methanol and incubated in toluene to facilitate the detachment of the polycarbonate filter from the insert before embedding in paraffin. Tissue sections (6‐μm‐thick) were prepared perpendicular to the filter using a microtome and mounted onto slides for analysis. Paraffin‐embedded tissues were stained using hematoxylin–eosin.

For immunofluorescence, nonspecific binding was blocked by incubating slides in PBS containing 0.2% BSA and 0.02% Triton X‐100 for 1 h, followed by incubation for 1 h at room temperature with the primary antibody. The used antibodies were anti‐keratin 10 (Ab9026, 1:100, Abcam), anti‐involucrin (I9018, 1:200, Sigma‐Aldrich), or anti‐loricrin (Ab176322, 1:100, Abcam). After washes in blocking solution, tissue sections were incubated with secondary donkey anti‐mouse IgG antibody Alexa Fluor 568 (A10037, 1:1000, Invitrogen) or goat anti‐rabbit IgG antibody Alexa Fluor 488 (A11008, 1:1000, Invitrogen) 1 h at room temperature; then nuclei were counterstained with DAPI for 10 min (D9542, Sigma) at 2 μg/mL.

Visualization of BrdU^+^ cells was performed on tissue sections deparaffinized and incubated for 30 min in 10 mM citrate buffer pH 6.0 at 95°C. DNA was denatured by incubation of the section for 30 min with 2 N HCl at 37°C and then neutralized using 0.1 M borax pH 8.5. Anti‐BrdU (347580, 1:50, BD Biosciences, Franklin Lakes, NJ, USA) was incubated in PBS containing 0.2% BSA for 1 h at room temperature. After washes in PBS, sections were incubated with goat anti‐mouse IgG antibody Alexa Fluor 488 (A11001, 1:1000, Invitrogen) for 1 h at room temperature.

All sections were mounted with coverslips using Dako Glycergel Mounting Medium (C0563, Dako) and images were acquired on a Celldiscoverer 7 microscope (Zeiss).

### 4.12 Clonogenic Assay and Treatment With Neutralizing Antibody

NHEKs at early passage were seeded at a density of 400 cells/cm^2^ per well in 24‐well plates. The next day, culture media was replaced by mixed with CM from NHDFs exposed or not (CTL) to UVB and transfected or not (UN) with siNT or siATF6α. CM was normalized to 5000 cells/mL using serum‐free BME and then diluted to a 70%–30% ratio with complete KGM‐2 medium. For neutralization experiments, 0.1 μg/mL of anti‐IL8 (AF‐208, R&D Sytems) or control IgG (31245, Invitrogen) at the same concentration was added as the instructor suggested a neutralization dose (ND50) of 0.1–0.5 μg/mL in the presence of 20 ng/mL recombinant human IL8. The media was changed daily for 3 days. Forty‐eight hours after the last change, media colonies were fixed and colored with crystal violet. Images of each well were acquired using the IncuCyte S3 Live‐Cell Analysis System (Sartorius, Göttingen, Germany).

### 4.13 Transcriptomic Analysis of Fibroblasts

Total RNA was isolated from NHDFs exposed or not (CTL) to UVB (*n* = 3) using the same procedure as above. RNA quality was assessed using a NanoDrop 2000 (Thermo Fisher Scientific). RNA integrity (RIN) was evaluated with an Agilent 2100 Bioanalyzer (G2938B, Agilent, Santa Clara, CA, USA). RNA‐seq was performed by the Giga's Genomics platform (GIGA Genomics, Liège, Belgium) using the Illumina RNA‐Seq workflow. In brief, mRNA sequencing libraries were generated using the Illumina mRNA Stranded Ligation Kit (Illumina, San Diego, CA), following the manufacturer's instructions. A NovaSeq6000 sequencer was used to generate 40 million PE reads of 2 × 150 bp per sample. Transcriptome assembly and gene annotation and mapping were performed using the FastQC version 0.11.9 to access the quality score distribution of the sequencing reads. The low‐quality reads (Phred score ≤ 30) were removed using Trimmomatic V0.39 prior to the analysis. Parameters in Trimmomatic were ILLUMINACLIP:TruSeq3‐PE.fa:2:30:10, LEADING:3, TRAILING:3, SLIDINGWINDOW:4:15, Scanning the reads with a 4‐base wide sliding window and cutting when the average quality per base drops below 15, and MINLEN:25. At end, reads less than 25 bases long were dropped after all previous steps. The remaining qualified reads were aligned to the GRCh38 version Genome sequence (primary assembly) using STAR, allowing two mismatches in the alignment for each read. GRCh38.p14 was used for genome indexing files. For counting of reads mapped to each feature (gene), we used HTSeq.qount by union mood. Differential Expression Analysis of the UVB vs. CTL dataset was performed using EdgeR R‐package (Robinson et al. 2010) with applied statistical cutoffs (FDR < 0.05, log_2_(FoldChange) ± log_2_(1.5)). Gene Ontology analysis, based on the DEGs list, was first conducted with published gene sets or retrieved gene lists from publicly available databases such as MSigDB (Liberzon et al. 2011) and GeneCards (Stelzer et al. 2016) (www.genecards.org), respectively. Further Gene Set Enrichment Analysis (GSEA) and Overrepresentation analyses (ORA) were achieved with “fgsea” R package (Korotkevich et al. 2021) and “ClusterProfiler” (Wu et al. 2021) R packages, by querying Reactome Pathway (Fabregat et al. 2018), MSigDB, KEGG, and DOSE (Yu et al. 2015) databases. In our analysis, a very small gene set (*n* < 9) has not been considered. The significance of GO terms or enriched pathways was determined according to the statistical cut‐offs from p‐adjusted value (*p*adj < 0.05) and Normalized Enrichment Score (NES ± 1.5), and then plotted using R.

The software versions used were R (4.3.2), edgeR (4.0.16), fgsea (1.28.0), clusterProfiler (4.10.1), DOSE (3.28.2). The fGSEA parameters wer: OrgDb = ‘org.Hs.eg.db’, nPerm = 50, minGSSize = 3, maxGSSize = 800, pvalueCutoff = 0.05. The ORA parameters were pvalueCutoff = 0.05, pAdjustMethod = “BH”, minGSSize = 5, maxGSSize = 500, qvalueCutoff = 0.2.

### 4.14 Quantifications

All images were processed using Fiji software (ImajeJ2).

For the quantification of LaminB1 and 53BP1 in human skin samples, it was analyzed for each condition using three independent donors, with 50–150 individual cells counted per donor.

For the quantification of the proportion of SA‐βgal^+^ cells, it was determined by counting a total of at least 300 cells per condition.

For the quantification of the proportion of EdU^+^ cells, it was determined by counting a total of at least 200 cells per condition.

For the quantification of actin, micrographies were processed using a generalist algorithm for cellular segmentation named Cellpose. Predictive masks were further analyzed using Fiji to assess the morphology of fibroblasts, employing Fiji shape descriptors such as cell circularity (4π*area/perimeter^2^) or cell roundness (4*area/(π*major_axis^2^)).

For quantification on RHE, the epidermal thickness was measured in BrightField micrographies by determining the distance between the top of the polycarbonate filter and the bottom of the cornified layer. Fifteen measurements were performed throughout the tissue section for each RHE. BrdU^+^ keratinocytes in RHE were calculated as ((number of BrdU^+^ cells/total number of cells in basal layer) *100) for each condition, as described in Frankart et al. (2012).

For quantification of colony formation, pictures of each well were obtained using the IncuCyte imaging system. The number of colonies (containing more than 20 cells) was counted. *p*‐values ≤ 0.05 were considered significant.

### 4.15 Data Plotting and Statistics

Statistical analyses were conducted using GraphPad Prism (GraphPad Software, La Jolla, CA, USA). Data used for statistics were obtained from at least three independent biological experiments. Results obtained from skin biopsy samples are expressed as mean ± SEM, while results obtained from cells and RHE are expressed as mean ± SD. All the statistical tests applied are specified in the figures' legends. Differences with a significance level of at least *p* < 0.05 were considered statistically significant.

## Author Contributions

**Joëlle Giroud:** conceptualization, methodology, validation, formal analysis, investigation, writing – original draft, writing – review and editing, visualization. **Pauline Delvaux:** validation, investigation. **Laura Carlier:** investigation. **Clémentine De Schutter:** investigation. **Nathalie Martin:** methodology, investigation, writing – review and editing. **Raphaël Rouget:** software, data curation, visualization, writing – review and editing. **Ayeh Bolouki:** software, data curation, resources, writing – review and editing. **Valérie De Glas:** investigation. **Inès Bouriez:** investigation. **Florent Bourdoux:** investigation. **Sophie Burteau:** validation. **Julien Théry:** resources. **Gauthier Decanter:** resources. **Nicolas Penel:** resources. **Yvan de Launoit:** funding acquisition. **Benjamin Ledoux:** resources. **Corinne Abbadie:** writing – review and Editing. **Yves Poumay:** resources; writing – review and editing. **Olivier Pluquet:** conceptualization; methodology; writing – original draft; writing – review and editing; visualization; supervision; project administration; funding acquisition. **Florence Debacq‐Chainiaux:** conceptualization; methodology; resources; writing – original draft; writing – review and editing; visualization; supervision; project administration; funding acquisition.

## Conflicts of Interest

The authors declare no conflicts of interest.

## Supporting information


Appendix S1.

## Data Availability

Transcriptomic data have been deposited in ENA. Accession: PRJEB75267; Secondary accession: ERP159856.
