# Supplementary material for: Targeting ATF6α Attenuates UVB‐Induced Senescence and Improves Skin Homeostasis by Regulating IL8 Expression
Source: Aging Cell. 2025 Apr 16;24(6):e70024. doi: 10.1111/acel.70024 (PMC12151886; doi:10.1111/acel.70024)
Supplement: Supplementary file 1 — Appendix S1. [file ACEL-24-e70024-s001.pdf]

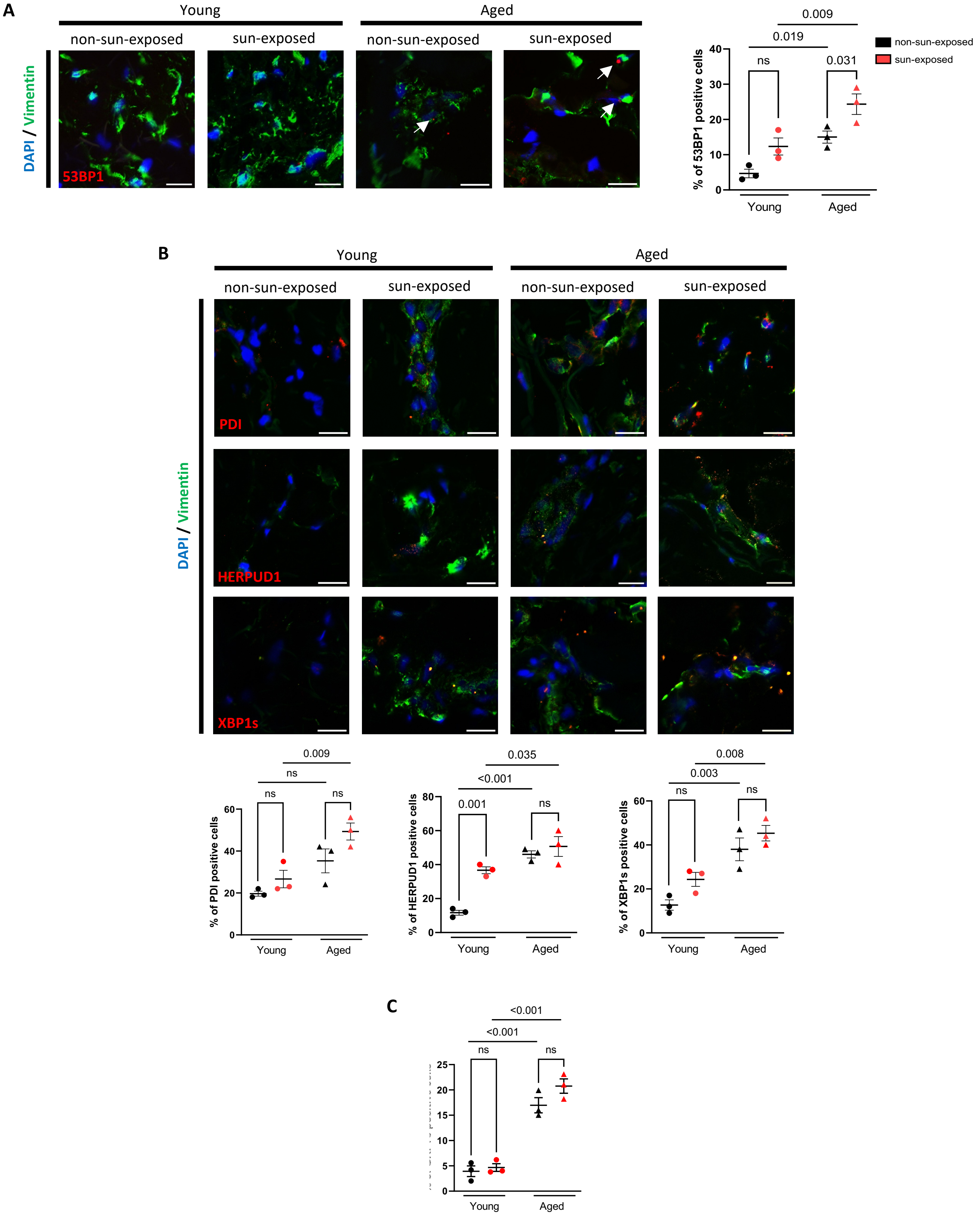

Figure S1. Aging and sun-exposition increase the level of 53BP1-positive cells and ER stress/UPR-related proteins in dermis sections.

A. (Left panel) Histological imaging of the dermis from both young (19-39 years old) and aged donors (56-82 years old) non-sun-exposed or sun-exposed. Samples were stained using immunofluorescence targeting 53BP1 (red). Vimentin (green) was used to delineate the dermal regions and DAPI (blue) for nuclei. Scale bar indicates 20 µm. (Right panel) Quantifications of the percentage of positive fibroblasts for 53BP1 determined by counting at least 100 cells per condition, (*n*=3). 53BP1 foci (red arrowheads) are pointed.
B. (Left panel) Samples were stained using immunofluorescence targeting HERPUD1, PDI, or XBP1s (red). Vimentin (green) was used to delineate the dermal regions and DAPI (blue) for nuclei. Scale bar indicates 20 µm. (Right panel) Quantifications of the percentage of positive fibroblasts for each protein of interest determined by counting at least 100 cells per condition, (*n*=3).
C. Quantifications of the percentage of positive fibroblasts determined by counting at least 100 cells per condition, (*n*=3).

Data information : Data in **(A-C)** are presented as means ± SEM of three donors per group. Statistical comparisons were performed using ANOVA2 followed by Šídák's multiple comparison tests. *p*-values shown represent statistical differences between non-sun-exposed (NSE) and sun-exposed (SE) dermis samples, and differences between young and aged group under the same sun exposure condition.

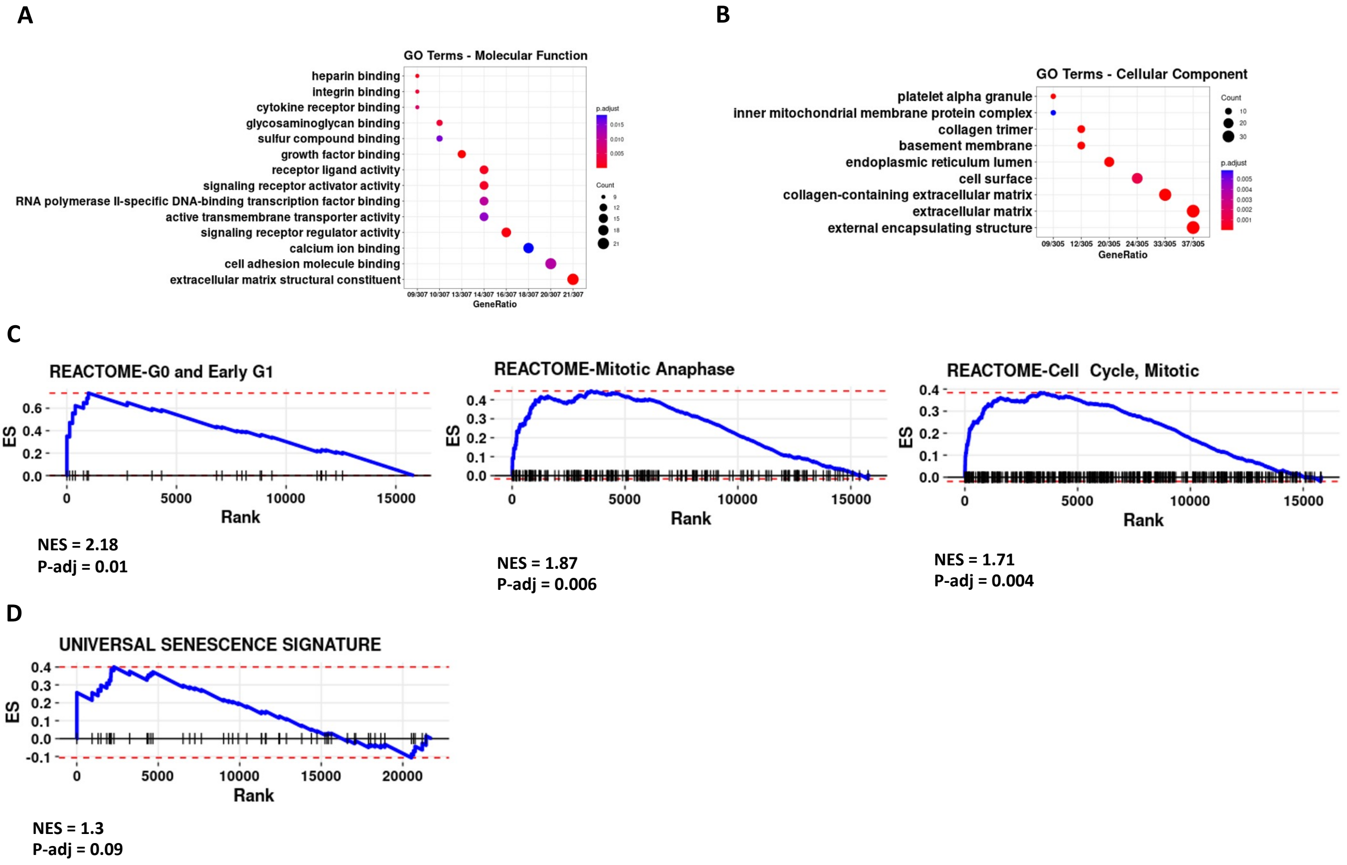

Figure S2. DEGs and transcriptome from UVB-induced premature senescent NHDFs display common senescence-related patterns.

NHDFs were exposed (UVB) or not (CTL) to 500 mJ/cm² UVB twice a day for 5 days. At three days after the last UVB exposure, mRNA was extracted.

A. ORA of Molecular Function (MF) GO terms of differentially expressed genes in UVB vs. CTL NHDFs. Gene ratio provides ratio of input DEGs that are annoted in MF terms.

B. ORA of Cellular component (CC) GO terms of differentially expressed genes in UVB vs. CTL NHDFs. Gene ratio provides ratio of input DEGs that are annoted in CC terms.

C. GSEA enrichment plot of the most positively enriched pathway associated with the whole UVB-induced senescent NHDFs using reactome database. (NES, normalised enrichment score).

D. GSEA enrichment plot of the most positively and negatively enriched pathway associated with the whole UVB-induced senescent NHDFs using Universal senescence signature published in *Hernandez-Segura et al.* 2017. (NES, normalised enrichment score).

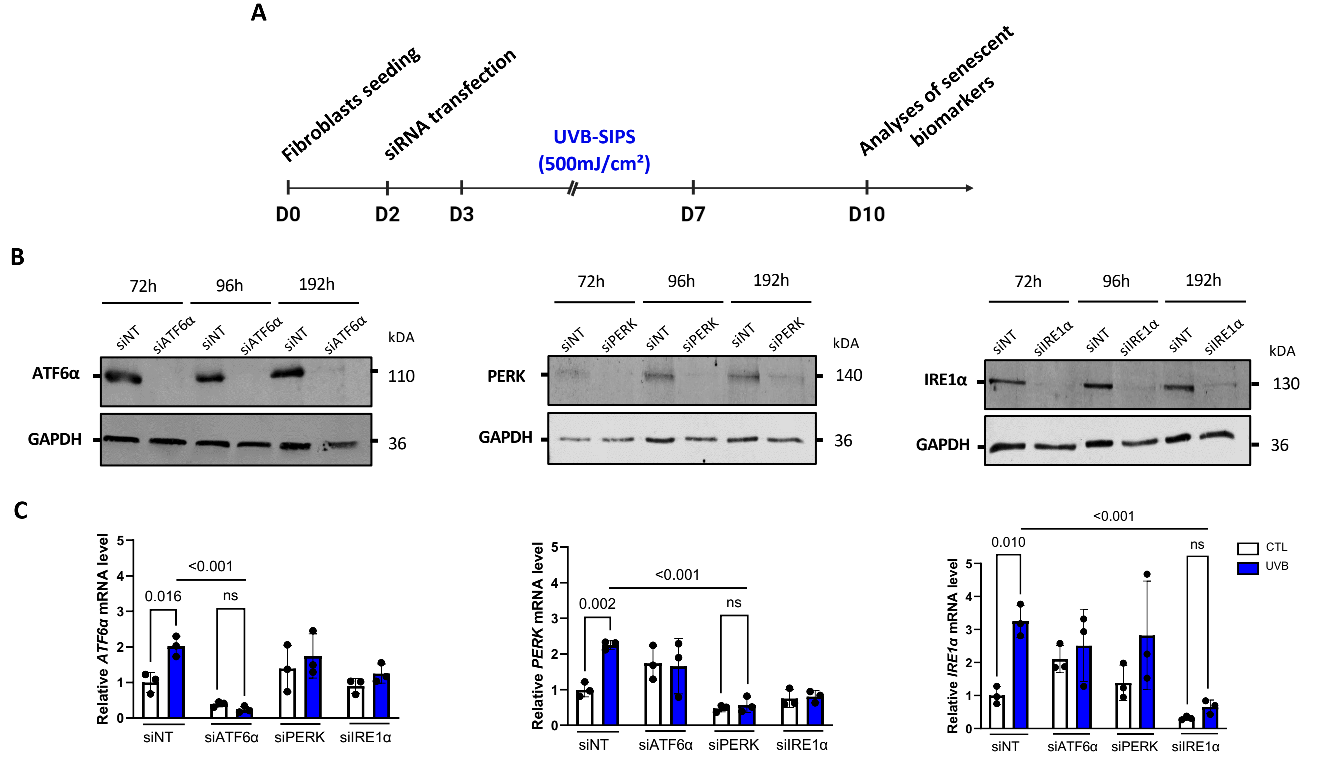

Figure S3. Validation of ATF6α, PERK, and IRE1α knockdown efficiency in NHDFs.

A. Schematic experimental RNA interference strategy for *in vitro* knockdown of the three UPR arms (ATF6α, PERK and IRE1α) in UVB-SIPS model. Details of the model are provided in Materiel and Methods.

B. NHDFs were transfected with non-targeting control siRNA (siNT) or with siATF6α, siPERK or siIRE1α. Representative Western Blots for ATF6α, PERK, and IRE1α at 72h, 96h, and 192h post transfection. GAPDH was used as loading control, (*n*=1).

C. NHDFs were transfected with non-targeting control siRNA (siNT) or with siATF6α, siPERK or siIRE1α 16 hours before repeated UVB exposures at 500 mJ/cm² twice a day for five consecutive days. Three days after the last UVB stress, mRNA was extracted, (*n*=3). Relative mRNA level of *ATF6α*, *PERK*, and *IRE1α* were quantified using RT-qPCR and were normalized to *RPL13A*. Results are expressed as ratio related to CTL siNT cells.

Data information : Data in (**C**) are presented as means ± SD. Statistical comparison was performed using ANOVA2 followed by Šídák's multiple comparaison tests. *p*-value shown represents differences between unexposed (CTL) and exposed (UVB) cells and differences between non-targeting control siRNA (siNT) and siATF6α, or siPERK, and siIRE1α.

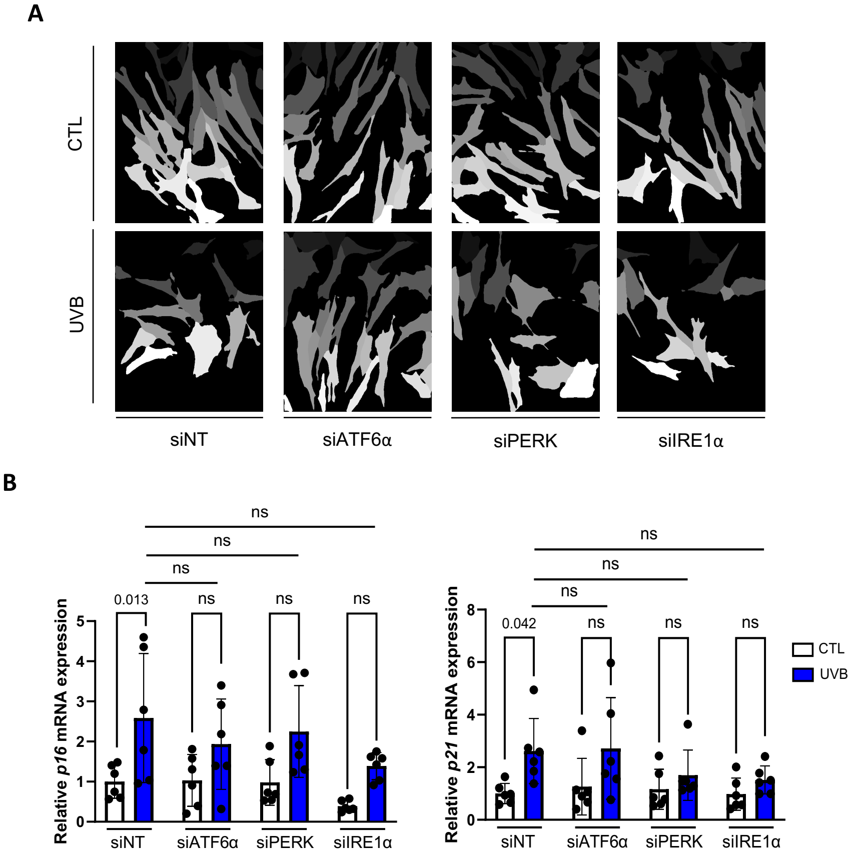

Figure S4. Impact of UPR invalidation before repeated UVB exposures on the senescent hallmarks.

NHDFs were transfected with non-targeting control siRNA (siNT) or with siATF6α, siPERK or siIRE1α 16 hours before repetitive UVB exposures at 500 mJ/cm² twice a day for five consecutive days.

A. Predicted mask of F-actin micrographies from the generalist algorithm for cellular segmentation, Cellpose. Masks have been generated to use shape description tools with Fiji.

B. Three days after the last UVB stress, mRNA was extracted, (*n*=6). Relative mRNA level of *p16*^{INK4A} *and p21* ^{WAF1} were quantified using RT-qPCR and were normalized to *RPL13A*. Results are expressed as ratio related to CTL siNT cells.

Data information : Data in (**B**) are presented as means ± SD. Statistical comparison was performed using ANOVA2 followed by Šídák's multiple comparaison tests. *p*-value shown represents differences between unexposed (CTL) and exposed (UVB) cells and differences between non-targeting control siRNA (siNT) and siATF6α , or siPERK, and siIRE1α.

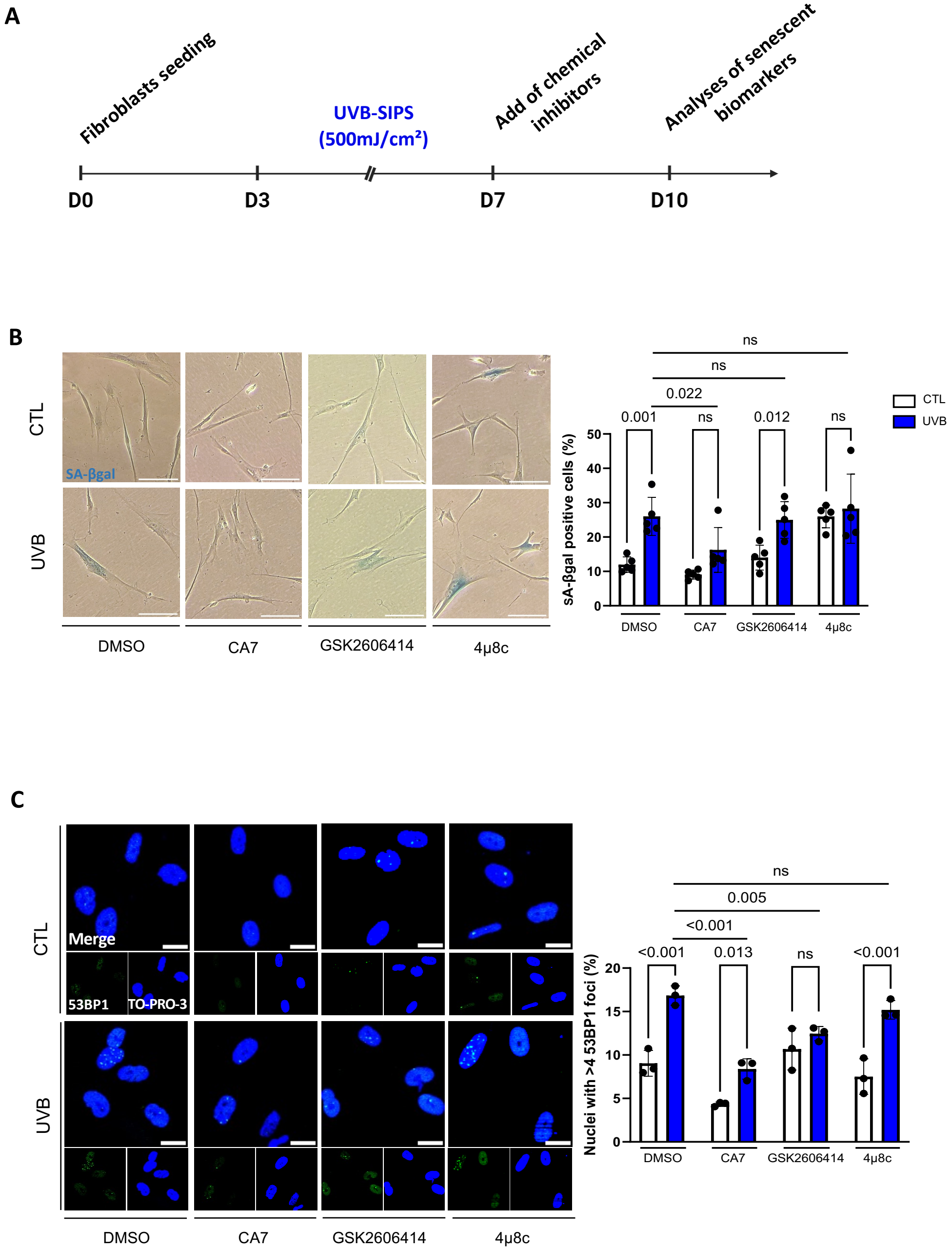

Figure S5. Inhibition of ATF6α after repeated UVB exposures prevents the complete establishment of the senescent phenotype.

AG04431 were repetitively exposed to 250 mJ/cm² of UVB, twice a day for five consecutive days. After the last UVB stress, chemical inhibitors of the ATF6α arm (CA7), PERK (GSK2606414) or IRE1α (4μ8c) were added. The biomarkers of senescence were studied three days after the last UVB exposure.
A. Schematic experimental strategy for *in vitro* inhibition of the three UPR arms in UVB-SIPS model. Details of the model are provided in Materiel and Methods.
B. (Left panel) Representative micrographies of SA-βgal staining (blue). Scale bar indicates 100 μm. (Right panel) Quantification of SA-βgal positive cells determined by counting 300 cells per conditions, (*n*=5).
C. (Left panel) Representative micrographies of 53BP1 staining (green) and TO-PRO-3 (blue). Scale bar indicates 20 μm. (Right panel) Quantification of the percentage of cells harboring more than four 53BP1 foci determined by counting 200 cells per conditions, (*n*=3).

Data information : Data in (**B-C**) are presented as means ± SD. Statistical comparison was performed using ANOVA2 followed by Šídák's multiple comparaison tests. *p*-value shown represents differences between unexposed (CTL) and exposed (UVB) cells and differences between control DMSO and CA7, or GSK2606414, and 4μ8c.

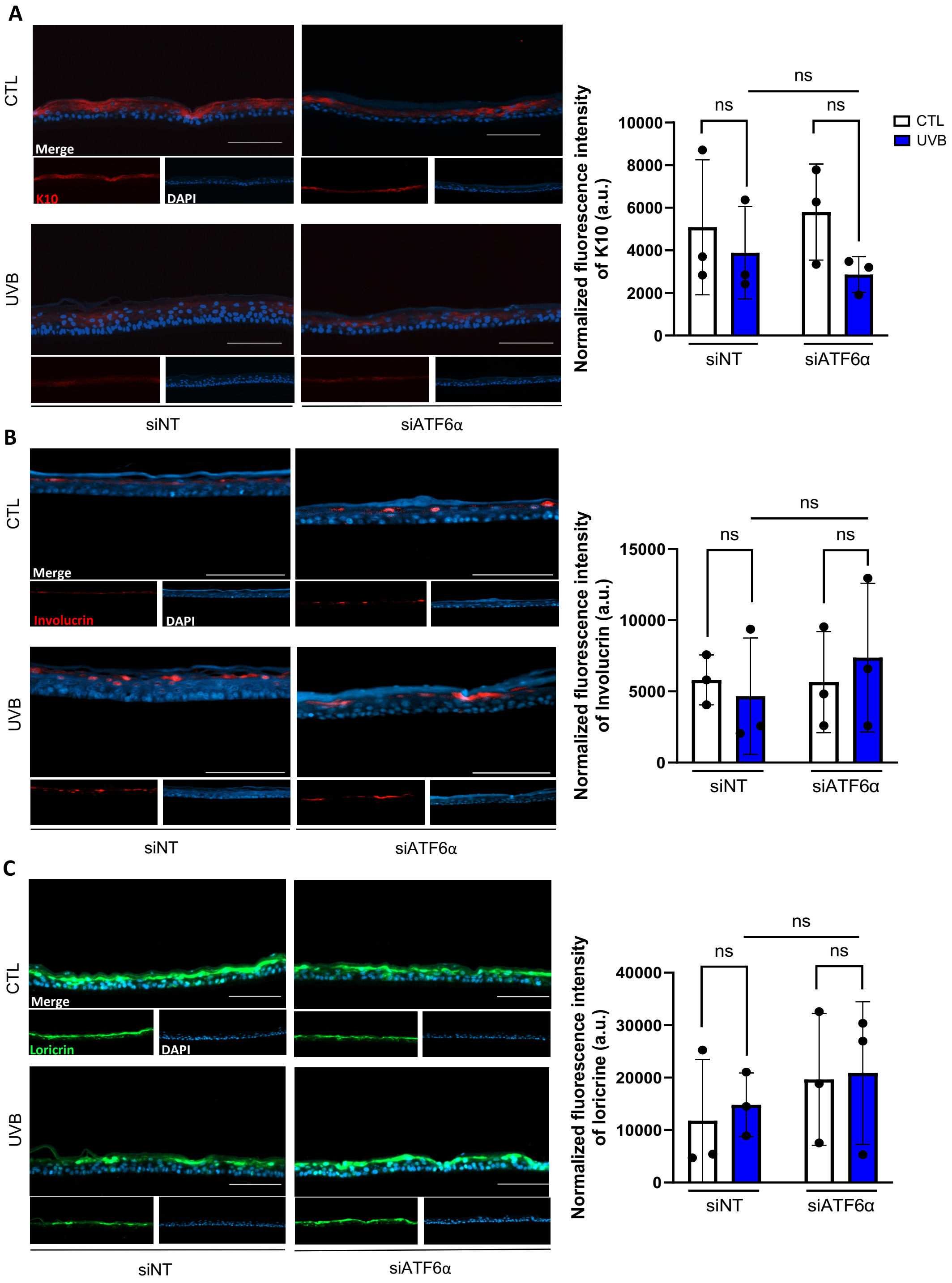

Figure S6. Conditioned media from fibroblasts do not impact keratinocytes differentiation in RHE.

RHE were grown using CM from NHDFs exposed or not (CTL) to UVB and transfected or not (siNT) with a siATF6α for 8 consecutive days.
A-C. (Left panels) RHE were stained using immunofluorescence targeting K10 (red) (**A**), involucrin (red) (**B**), or loricrin (green) (**C**). DAPI (blue) was used to stain nuclei. Scale bar indicates 100 µm. (Right panels) Quantifications of normalized fluorescence intensity.

Data information : Data in (**A-C**) are presented as means ± SD. Statistical comparison was performed by paired *t*-test. *p*-value shown represents difference between unexposed (CTL) and exposed (UVB) cells, and differences between UVB siNT and UVB siATF6α.
